# Evaluating the Irritant Factors of Silicone and Hydrocolloid Skin Contact Adhesives Using Trans-Epidermal Water Loss, Protein Stripping, Erythema, and Ease of Removal

**DOI:** 10.1021/acsabm.3c00874

**Published:** 2023-12-27

**Authors:** Edward Dyson, Stephen Sikkink, Davide Nocita, Peter Twigg, Gill Westgate, Thomas Swift

**Affiliations:** † School of Chemistry and Biosciences, University of Bradford, Bradford BD7 1DP, U.K.; ‡ Faculty of Engineering and Informatics, University of Bradford, Bradford BD7 1DP, U.K.

**Keywords:** silicone, adhesive, skin contact, vapor permeability, TEWL, peel strength

## Abstract

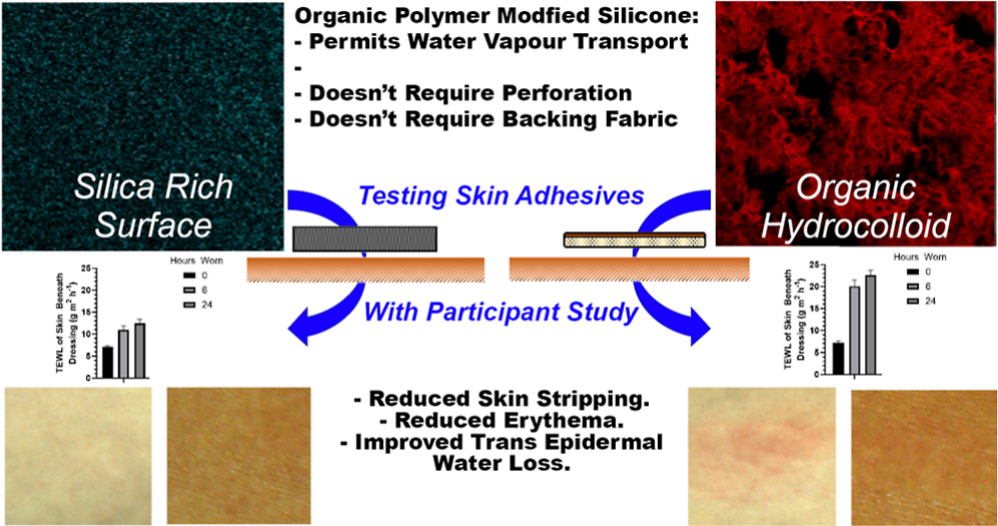

A composite silicone skin adhesive material was designed to improve its water vapor permeability to offer advantages to wearer comfort compared to existing skin adhesive dressings available (including perforated silicone and hydrocolloid products). The chemical and mechanical properties of this novel dressing were analyzed to show that it has a high creep compliance, offering anisotropic elasticity that is likely to place less stress on the skin. A participant study was carried out in which 31 participants wore a novel silicone skin adhesive (Sil2) and a hydrocolloid competitor and were monitored for physiological response to the dressings. Trans-epidermal water loss (TEWL) was measured pre- and postwear to determine impairment of skin barrier function. Sil2 exhibited a higher vapor permeability than the hydrocolloid dressings during wear. Peel strength measurements and dye counter staining of the removed dressings showed that the hydrocolloid had a higher adhesion to the participants’ skin, resulting in a greater removal of proteins from the stratum corneum and a higher pain rating from participants on removal. Once the dressings were removed, TEWL of the participants skin beneath the Sil2 was close to normal in comparison to the hydrocolloid dressings that showed an increase in skin TEWL, indicating that the skin had been highly occluded. Analysis of the skin immediately after removal showed a higher incidence of erythema following application of hydrocolloid dressings (>60%) compared to Sil2, (<30%). In summary, this modified silicone formulation demonstrates superior skin protection properties compared to hydrocolloid dressings and is more suitable for use as a skin adhesive.

## Introduction

The damage to skin following the use and removal of adhesive dressings from skin is not solely down to the peel force of the material.^1^ Moisture control has long been known to be a defining factor in the role that dressings play in successful wound healing.^2^ Balancing hydration in the skin is a delicate process and, if breached, can result in maceration and breakdown of the skin barrier and exposure of subepidermal structures. Despite that, for damaged skin, retaining moisture has been shown to be beneficial for repair—and there is a distinct difference between wound hydration and maceration although they can present similar dermatological responses.^3^

Occlusive materials placed over the skin can influence skin moisture content either via their absorptive capacity or their water vapor permeability, and so a range of different materials are recommended for skin adhesive dressings depending on the medical needs required.^4^ Depending on the need for moisture retention, light perforation still permits air and vapor permeability while forming an effective waterproof barrier against infection. Medical dressings are an essential component of wound healing, as although the majority of wounds heal without complication, acute and chronic wounds can require extensive therapeutic intervention.^5^ Dressings act as a protective barrier to shield damaged or irritated skin, isolating the wound from external dirt/microbial contamination, maintaining a high internal humidity ideally while encouraging gaseous exchange to encourage natural healing. There has been investigation into the use of dressings as a therapeutic intervention, and much research has been done on introducing accelerants to encourage wound healing through the dressing material.^6^ As such, dressings are an essential, sometimes overlooked, component of medical treatment with a high impact on patient outcomes.^7^ However, in order to shield and protect wounds, dressings also coat healthy skin, which runs the risk of causing patient discomfort, largely due to occlusion of water transmission or retention of wound exudate.

There are many different technologies that allow for skin adhesion—this is a challenging technological area because the occlusion resultant from close contact with the skin causes a physiological response within hours.^8^ Prolonged exposure to humid environments is a clear factor in a range of dermatological conditions due to its impact on trans-epidermal water loss (TEWL).^9^ Despite that, our need to adhere materials to the skin surface for wound dressings,^10^ wearable technology,^11^ cosmetic use,^12^ or other medical interventions means as new materials and products enter this market, it is important to analyze their impact on skin structure and health. As a result, there has been much interest in the development of perspiration simulators for evaluating the performance of adhesives under realistic wear conditions^13^ and further studies to improve our understanding of how moisture management in occlusive adherent materials impacts skin health.

Recently, Trio Healthcare launched Sil2 silicone technology, a modified silicone product that is designed to offer improved breathability over conventional adhesive flanges used in the treatment of ostomates.^14^ The ostomy market is a particularly challenging area of medical treatment for adherent dressings that presents unique challenges to dermatologists.^15^ Silicone as a material has had plenty of prior application in this field due to its flexible, nontoxic, adhesive properties. Unlike most silicone products on the market, however, Sil2 is hydrophilically modified via the incorporation of water-absorbent polymer additives to increase the potential for moisture vapor diffusion^16^ meaning it is designed to offer a less-occlusive surface than other technologies.^17^ This has been shown to be of benefit for stoma patients, reducing peristomal skin complications and improving their quality of life.^18^ Other studies have added polymer additives such as poly(methyl methacrylate) to alter the mechanical properties of silicone rubbers to make them suitable for maxillofacial prosthesis^19^—however, to our knowledge, this is the most complete study of a novel silicone-composite product.

We are interested in the new adhesive skin-contact material fundamental properties to reduce skin irritancy in Sil2, which we hypothesize is primarily due to its low adhesion and high vapor permeability but may also see contributions due to its high elasticity. During the time in contact with human tissue (as well as during the application and the removal of the patches), the materials are subject to composite stresses, which, because of the adhesive characteristics at the interface, can be transferred to the skin. Therefore, to better understand the viscoelastic behavior of the dressing materials, especially in terms of creep response, dynamic mechanical analysis (DMA), and shear rheology, tests were carried out to characterize the compounds under tensile and shear stress fields, respectively. It should be noted that evaluation of skin adhesives does not have clear international standards at this point in time, although there are set test methods to determine peel or stripping strength of adhesive bonds and biological evaluation of medical devices, which were carefully considered in the design of experiments for this study.

This study is designed to explore whether the new Sil2 technology really does provide those benefits by testing the skin response to prolonged wear of the new silicone material in healthy volunteers in comparison to hydrocolloid alternatives. We have tested this novel material against abdominal and forearm tissue to provide greater insight into potential skin–material interactions of this wearable adhesive technology. We also discuss the role of TEWL in discussing skin health and function and the value of measuring TEWL within our experiments.

## Review of the Stratum Corneum, Trans Epidermal Water Loss, and the Need for Less-Occlusive Dressings

The outermost component of the skin, the stratum corneum (SC), is composed of enucleated and flattened corneocytes, formed from terminal differentiation of epidermal keratinocytes, surrounded by lamellae sheets enriched with free fatty acids and ceramides.^20^ There is a steady flux of water through the skin as condensed water diffuses from the extremely hydrated layers of the epidermis and dermis to the upper layers of the SC.^21^ The thickness of the SC is around 10–20 μm, and this variability is one of the main factors in varying liquid flux, alongside the size of the corneocytes, external temperature, and pressure. The SC has many protective functions, one of the most crucial ones being the permeability barrier which ensures that the body remains water-tight and allows survival in very dry environments.^22^ Usually, the healthier the skin protective coat, the slower the diffusion of water across the SC.^23^ Water exits the skin via two methods; either via a trans-epidermal route or via sweat glands. If the skin is occluded, then this diffusion is prevented and the skin is over hydrated. When the occlusion is removed, then the accumulated water evaporates which shows a higher TEWL rate than the SC’s primary value.^24^

Recent studies show when skin is damaged, e.g., by deep burns, there is incomplete re-epithelialization of the epidermis due to an absence of a stem cell reservoir; therefore, a less-functional epidermis increases TEWL and reduces moisture retention. Because of this, patients experience an increased likelihood of scarring and itching, resulting in inflamed and vulnerable skin.^25^ Damaged SC is also more vulnerable to infection and disease, a risk factor increased in patients subject to repeated application of dressings where adhesive films are pushed onto the skin and then removed repeatedly. These superficial layers, including corneocytes, of the SC stick to the adhesive film and can be recovered and tested further.^26^ Skin damage occurs when skin-to-adhesive attachment is greater than skin-to-skin attachment, which correspondingly allows separation of the epidermal layers, known as medical adhesive-related skin injury.^27^ As well as this, there is also moisture-related skin damage, which is caused by extended exposure to different sources of moisture such as stool, urine, sweat, and wound exudate. Skin maceration can occur because of low oxygen permeability due to skin contact dressings which occlude TEWL.^28^

Currently, a range of fluid-trapping dressing materials are available on the market,^7^ but the current dominant products are hydrocolloids. These interact with skin tissue and adhere quite strongly to periwound skin, forming a gel near the wound. Pectin, gelatin, and sodium carboxymethylcellulose (CMC) in the hydrocolloid enable the formation of the gel, and adherence to skin is provided by a tackifying agent. As the hydrocolloid becomes more hydrated, there is higher adhesion; however, this also saturates the skin which can cause maceration if left for an extended period of time. Hydrocolloid dressings can also take up wound exudate, which can give rise to leakage and malodor which can have a negative impact on patients’ quality of life.^29^ To combat these negative attributes, alternative dressing technologies have been proposed, and silicone materials have shown to offer several advantages compared to hydrocolloids.^30,31^ Here, the level of adhesion remains the same throughout the duration of the wear time and does not increase with skin saturation; additionally, silicone dressings are associated with significantly less damage to SC compared to hydrocolloids.^32^ It has been proposed that silicone-based products, e.g., gels and sheets, are recommended as the noninvasive, gold-standard option for the treatment and prevention of scars.^33,34^ The configuration of the silicone sheets differs widely, with some containing only medical-grade silicone and others containing a mixture with polymer additives providing extra reinforcement, allowing the product to be thin, flexible, and breathable. Silicone sheets can be self-adhesive or require tape to attach them to the skin.^35^

TEWL is a measure of the amount of water transiting out of the skin under (advisedly) standardized conditions of temperature and humidity in the environment of the subject. It is a measure of skin health and can be measured using open and closed chamber methods. Water loss from skin has two components; one is transepidermal—so-called insensible water loss, and the other is via sweat glands, which are under autonomic control. Trans-epidermal water loss is influenced by factors such as thickness of the stratum corneum (lower in thicker sites); size of corneocytes (larger TEWL for smaller corneocyte size); local tissue temperature (TEWL is larger when tissue is warmer); and boundary layer water vapor pressure (TEWL is higher when humidity is lower).^23^ TEWL is variable across the body, with higher levels in feet and palms, but forearm and abdomen have similar TEWL values, which helps justify the use of forearm as a model site.^36^ Sweat gland distribution literature has been reviewed by Taylor and Machado-Moreira,^23^ and Table 1 in this review shows that abdomen and forearm have similar sweat gland densities, supporting the forearm as a model site for the evaluation of new adhesive materials Clothing and other occlusive materials will increase the boundary layer water vapor pressure, which could be a factor for users of the adhesive products and dictates that a period of acclimatization is required before TEWL is measured.

**Table 1:** Comparison of Body Site/Skin Damage TEWL Values from the Literature^f^

study^a^	temp (°C)/humidity (%)	arms	torso	stripped arm^b^	stripped torso^b^
Gao^26^	18–22/45–55	2.23 ± 1.24 (60)	3.04 ± 2.29 (30)	8.25 ± 4.75 (60)	20.06 ± 12.71 (30)
Fluhr^47^ (VP)^c^	22–23/44–52	5.57 ± 1.17 (11)		21.52 ± 1.17 (11)	
Fluhr^47^ (210)^d^	22–23/44–52	6.44 ± 0.48 (11)		18.95 ± 1.17 (11)	
Fluhr^47^ (300)^e^	22–23/44–52	12.17 ± 0.85 (11)		22.09 ± 3.21 (11)	
Luebberding^50^^(F)^	20/50	9.10 ± 2.25 (150)	9.25 ± 2.62 (150)		
Luebberding^50^^(M)^	20/50	5.50 ± 2.02 (150)	6.96 ± 3.01 (150)		
Grove^48^	20/<50	3.73 ± 1.19 (28)		6.85 ± 4.97 (28)	
Clausen^54^	unknown	5.90 ± 1.36 (5)			
Döge^51^*	20/30		6.96 ± 0.68 (8)		34.32 ± 7.07 (6)

a This shows the study reference where full details of the methodology can be found.

b Stripped indicate that this measurement was carried out after an adhesive patch was applied and removed to study potential damage to the stratum corneum. We note that the type and strength of the adhesive, and the number of times it is applied and removed, are variable across the listed studies.

c Results from Fluhr et al. are separated by a vapometer.

d Results from Fluhr et al. are separated by a TM210 instrument.

e Results from Fluhr et al. are separated by a TM300 instrument.

f Data is presented as X ± SD (N) for each respective study. ^(F)(M)^Study reports exclusively on female (F) or male (M) participants. *This study is conducted on ex-vivo abdominal tissues and provided for reference.

One question of interest is what happens to the rate of water loss from the skin when it is occluded or wet. This is relevant in situations where an adhesive can absorb water such that the boundary layer becomes saturated. It is known that stratum corneum can become saturated and has the capacity to absorb a significant amount of water —300–400% dry weight^37^ when exposed to water which is mildly disruptive to the structure of the barrier.^38^ Unless the SC is damaged, then this water content will return to normal. Prolonged occlusion/saturation is an important factor in wound care.^39^

In disrupted (irritated or mechanically damaged) skin, TEWL is higher. Mild disruption to the SC barrier induces repair mechanisms and semiocclusion can help restore barrier function.^40,41^ Significant occlusion of the skin will reduce TEWL but leave the SC subject to excessive water content, leading to the risk of maceration. Normal skin does not seem to be adversely affected by occlusion for short periods, even with repeated occlusion, but occlusion over longer or sustained periods together with mild skin irritation may adversely affect barrier function.^42,43^ A recent review comparing several types of semiocclusive silicone products for scar reversal using TEWL and SC moisture levels as measured end points also suggests that products that can reduce TEWL to near normal values help skin recover.^44^ This could be of value when considering the relative occlusive properties of silicone adhesive formulations under test. Some differentiation on the selection of product properties depending on the status of the skin might also be important to ensure healing and maintaining health. Scar tissue also has a higher TEWL, and patients with stretch marks at the affected site (e.g., in ostomates) might have altered barrier properties that could be impacted by occlusion.^45^ From this examination of the literature, recommended procedures required for TEWL measurement involve a short acclimation period in a room with measurable room humidity (RH) and temperature with these being less than 50% RH and around 20 °C.

The results described in Table 1 suggest that TEWL values of normal skin, whether using open- or closed-type systems, are generally between 2 and 10 g^–2^ h^–1^ in vivo and also in ex vivo skin models. Procedures known to damage the skin generate a much higher TEWL value above 10 g^–2^ h^–1^ (tape stripping and detergents), especially using ex vivo skin. Skin that is damaged or has the propensity to be damaged (nonlesional and lesional skin in eczema patients) and scar tissue also had a higher-than-normal TEWL.

Examination of the literature on sweating and water retention by the stratum corneum suggests that prolonged occlusion of the skin influences corneum barrier function through swelling.^38^ It would appear to be a factor in situations where dressings are required and prolonged hydration of the skin delays healing.^39^ However, the relationship between skin occlusion/100% skin saturation and sweat gland activity is complex as it is also related to control of body temperature. Given that the density of sweat glands on the abdomen is in the lower range, the functionality of sweat glands under adhesive materials that are not occlusive may not be affected. This would need a further detailed examination of the literature and is beyond the scope of this short review. To answer the question “when skin is occluded, does the skin underneath the occluded zone still produce moisture, or does the body compensate in other ways?” requires an understanding of the level of occlusion, effects on tissue warmth, and whether the effects are prolonged.

A range of experimental devices to study TEWL, including the vapometer used in this study, are well utilized in clinical practice and have been compared by Klotz et al.^46^ We have identified in Table 1 where equivalent, or alternative, instrumentations have been used to indicate a normal distribution of TEWL against the body site within the literature. Depending on the device used, the exact measured value of a skin site can vary as much as from 2 to 12 g^–2^ h^–1^, but each device is generally internally consistent.^47^ This variability means that providing an average TEWL for healthy individuals depends on the instrumentation employed—but across the full literature sampled, we found that healthy human TEWL could be assumed to fit between this range and that any level of tape stripping would increase this value by a significant portion.^26,47,48^ Different studies have reported that TEWL varies more significantly with body site and gender than with age.^49,50^ Although many medical tests have been performed in vitro, ex vivo studies are possible and can provide comparable values whose TEWL matches living skin tissues^51^ but is complicated by the need to assess preserved skin integrity post thawing.^52^ One variable rarely accounted for is the time of measurement as further data has shown that TEWL varies with the circadian rhythm of the individual.^53^ These data are summarized in Table 1 which shows the variation in average TEWL for healthy skin across arms and torso. Each study has a variation in experimental design—with the number of times the adhesive is removed and over which time period being variable—but it shows clearly both the trends and variance of data across the literature.

Analysis of the data from studies that compare both torso and forearms suggests similarity in the TEWL values of the two body sites. However, there was a difference in TEWL following tape stripping, suggesting differences in the resilience of the stratum corneum.^26^ Due to the normal location of the adhesive patches beneath layers of clothing, the relative humidity and external exposure of adhesives in these locations will vary during the wear period. As we were investigating adhesives in peel testing on the forearm, it was decided to proceed with the study using both body sites to get the maximum amount of information regarding adhesive/skin interactions but treat the data independently to ensure full transparency of our findings.

## Materials and Methods

Adhesive dressings including silicone dressings (Sil2, Trio healthcare), silicone-coated polyurethane foam (Mepilex), and hydrocolloids (Salts Healthcare hydrocolloid flange extenders, Eurotec Varimate hydrocolloids) were analyzed as a part of this study. All were used in initial material evaluation, but only Sil2 and Salts Healthcare was advanced to be used in the full participation study.

Scanning electron microscopy (SEM) was carried out on an FEI Quanta 400 instrument with an Oxford Aztec EDS attachment to determine elemental composition of materials. Samples were attached to adhesive stubs without any coating to provide a high-resolution analysis of material surfaces. Fourier-transform infrared spectroscopy analysis of adhesive surfaces was carried out on a LUMOS II FT-IR microscope. Biocompatibility measurements were carried out using human dermal fibroblast cells isolated and cultured from donor healthy normal human haired scalp (Female, 45y) as a part of an elective (cosmetic) plastic surgery operation. Dermal fibroblasts (DFs) were isolated from the papillary dermis by cell outgrowth after the removal of epidermis. Primary DF cells were expanded in vitro in Dulbecco’s modified Eagle medium supplemented with 10% fetal bovine serum, 2 mM Glutamax, and 1× pen/strep (all Gibco Thermo Fisher, UK) with feeding at regular intervals. For details of full biocompatibility measurements, please see Section S3 (Figures S8 and S9).

Uniaxial mechanical measurements were carried out on an Instron 5568 instrument equipped with a 100 N load cell and standard small tensile grips at 200 mm/min crosshead speeds (test designed to match the ISO 37 2017 standard using type-3 specimens). Samples were analyzed up to five times to produce stress/strain curves and determine elastic modulus and break points. DMA tests were performed with a TA Instruments Q800 model using tensile grips: after an isothermal step (5 min) at the test temperature (28, 32.5, and 37 °C), rectangular samples of 16 and 10 mm of length and width, respectively, were displaced under a constant tensile stress of 3 kPa for 10 min, followed by a 20 min recovery time. Shear creep tests were performed with an Anton Paar Physica MCR 501 rheometer equipped with a parallel plate geometry and a Peltier plate: after an isothermal step (5 min) at the test temperature (28, 32.5, and 37 °C), disks of 25 mm diameter were displaced under a constant shear stress of 500 Pa for 10 min, followed by a 20 min recovery time. Amplitude sweep test was also carried out on Sil2 at 32.5 °C. A disk of 25 mm diameter was subjected to a logarithmic strain ramp from 0.1 to 100% in oscillatory mode (ω = 10 rad/s), and the resulting shear stress was plotted against the shear strain.

To test moisture response, samples were initially analyzed in a sandwich design, with the adhesive contained between two barrier sheets (PU film above—to mimic the protective layer of a medical dressing and Whatman filter paper below). Beneath this, a piece of tissue paper was placed, either wet or dry. Samples were placed in an oven and incubated at variable temperatures and relative humidity for 24 h to ensure that they were fully acclimatized to their environment. After this, the samples were removed and the vapor permeability (VP) was measured through the top PU film across four different points on each surface, and each measurement was repeated four times, providing an *n* = 16 to determine sample variation. In the presence of wet tissues, the humidity of the oven was measured to be 45%. Swelling measurements were carried out by cutting 10 × 15 mm (approximately) strips of each adhesive material. Six samples of each dressing were separately stored within a Corning 24-well plate with a unique identifier. Each well was imaged using a Bysameyee HD 2MP microscope with 40× magnification. Image-J software was used to remove the background of the image, and then, the dimensions of the sheet were determined. Strip swelling potential was determined as % change in area of the flat strip before and after 24 h immersion in aqueous suspension. Some samples decomposed on swelling resulting in a reduced n for that material—although this indicates material instability to high levels of water content. Further details of both VP and swelling % are shown in the Supporting Information (Section S1).

The participant study was carried out with 32 participants (15 M and 16 F), 18 of which wore the patches for approximately 6 h (a single working day) (8 M and 10 F) and 14 of which wore the patches for 24 h (8 M and 6 F). All volunteers provided written informed consent prior to participation in the study. Participants varied in age from 19 to 58, with an average median of 29. Participants stated their ethnicity (25 White, 3 Arabic, 1 Black, and 2 Asian). Seven participants (4 F and 3 M) indicated in the survey after the study was completed that they had some minor history of eczema (either lapsed issues in childhood, historical psoriasis, or one example of site-specific discoid eczema). Some participants (5 M and 5 F) consented to the skin sites the patches were applied to be captured as a 3D surface using a Vectra H1 XP camera for accurate before and post study comparisons. The study applied adhesive dressings to both the upper forearms and lower torsos of participants. Before TEWL measurements were made, participants acclimatized in a controlled environment for 20 min at rest, before measurements were made and/or adhesive strips were applied/removed. When adhesive strips were removed, the TEWL measurements were made within 5 min of removal. Three initial TEWL meter readings were recorded using a Delphin Technologies VapoMeter, comparing the TEWL across each dressing and a third control site on bare skin. Patches were applied under controlled conditions with constant room temperature and humidity (23.6 °C, 36% room humidity—measured using an Ebro Room Climate Monitor RM100). The participants were then asked to carry out their normal daily routines for an allotted time period (6 h/24 h) and returned to have the patches removed. Four candidates (3 F and 1 M) reported high levels of activity (i.e., gym visits, running, and swimming), but the majority either had only minor activity (9 M and 8 F) or that they had been predominately inactive during the test (5 F and 4 M—all following 6 h wear). Twenty of the applicants reported that one or more of the patches had lifted during the course of the study; however, the vast majority of patches (223/248) remained in place. TEWL measurements were taken of the patches and a control measurement of the skin adjacent to each test area. Patches were removed and placed in sterile Petri dishes for further analysis, and the majority of candidates had the skin beneath the patches analyzed to measure the TEWL of skin after wear (8 M and 10 F) and consenting participants had the skin photographed to record any discoloration in reaction to the dressings. Participants were then asked to indicate the relative pain levels of removal of the silica or hydrocolloid dressings on a scale of 1–5 and report any other complications (such as itching).

Five Participants (3 F and 2 M) agreed to wear additional patches for 6 h which were removed via an in vivo 90° peel test using a Bio momentum Mach 1 system with a 10 kgf single-axis load cell. The dressing was on the patient’s forearms, which was positioned horizontally below the Mach 1 crosshead, with the dressing peeled from the top of the forearm toward the elbow. The distal edge of the dressing was clamped in the Mach 1 tensile grips such that the peel-front was on the tensile axis at zero displacement. The crosshead was then raised at 2 mm s^–1^ under displacement control to a maximum of 80 mm. The load was recorded throughout the test and analyzed in terms of the peak load required for adhesive failure and the steady-state peel load. Data was analyzed to determine Cohen’s *d* effect size analysis between two means.^55^

Silicone and hydrocolloid dressings removed from participants were stained using a QC Colloidal Coomassie. The dressings were stained for a 1 h incubation using the dye (20 mL per Petri dish) and then destained using distilled water (20 mL), while samples were gently rocked on a shaker plate (50 rpm). The plates were imaged using a ChemiDoc MP Imaging System to measure the intensity of the dyes at each blot. Inverted gray-scale histogram of all pixels from the images (0–255 intensity) were produced, and the mean value for each dressing was used to determine the difference in stain intensity. Images were processed against blank controls (stained but with no protein present), and the absolute differences between intensity were used to determine protein loading.

Three-Dimensional polarized light images of the participant’s skin were analyzed using Cloud Compare Open source software to project the textured skin surface onto a three-dimensional object. Direct comparison of the same area of skin both before and after the patch was applied, and all images were assessed for signs of skin damage, either due to reddening or detexturization of the surface.

## Results

The manufacturing of novel permeability-modified silicone wafers (Trio Sil2) is described previously.^16^ In brief summary, the skin-compatible silicone polymer network was derived via a two-step process—curing a vinyl-functionalized siloxane polymer and a silicon hydride containing a cross-linker in the presence of a metal catalyst. A superabsorbent polymer particulate (average particle size <150 μm) was distributed through the polymer network to absorb moisture, and additional permeability-modifying polymer was distributed within the polymer network. This material was previously tested for biocompatibility and incorporated into medical devices as a stoma baseplate following a clinical study to assess its impact on quality of life.^18^

A selection of flange adhesive materials was selected for initial screening, and these adhesive patches were analyzed in isolation before being considered for participant testing. These were the novel silicone (Trio Sil2), two hydrocolloid adhesives (Eurotec Varimate and Salts 0303), and an existing silicone adhesive wound dressing (Mepilex Border Lite). These materials had a range of thicknesses and swelling responses when immersed in liquid as described in Table 2. The modified silicone had the second highest VP, although as the Salts hydrocolloid is less than a third of the product thickness, it will offer significantly less resistance to gaseous diffusion. The silicone showed the smallest response to hydration as all other products demonstrated significant changes in dimensions either by contracting or expanding in the *x*–*y* dimensions when immersed in liquid over a 24 h period. If the measured vapor permeability of the materials was normalized against the product thickness, then the estimated permeability of the hydrocolloids per millimeter of the material was 7.3 and 1.9 g m^2^ h^–1^, while those of the silicone products were 4.9 and 16.9 g m^2^ h^–1^. The silicone products not only demonstrated a higher vapor permeability, but when the humidity of the environment was increased, their permeability increased, while the hydrocolloids decreased. Full details of this analysis are contained in the Supporting Information (see Sections S1 and S2).

**Table 2:** Adhesive Flange Materials Tested during This Study

product	material^a^	structure^b^	thickness (mm)^c^	swelling [% ± SD (*n*)]	dry VP^d^ (g m^2^ h^–^^1^)	moist VP^d^ (g m^2^ h^–^^1^)
Sil2	hydrophilicly-modified silicone gel	wrinkled sheet	0.904 ± 0.110	97.6 ± 4.6 (6)	5.46 ± 1.06	10.30 ± 1.54
Mepilex border lite	polyurethane foam with silicone adhesive layer	perforated sheet	3.740 ± 0.632	115.7 ± 3.9 (4)	4.53 ± 0.31	9.70 ± 0.20
Eurotec Varimate	hydrocolloid (pectin, gelatin, sodium CMC and polyisobutylene, latex, and synthetic elastomer free)	porous wafer	1.858 ± 0.221	113.8 ± 3.6 (6)	3.93 ± 0.85	0.53 ± 0.76
Salts 0303	hydrocolloid [CMC (Cecol) and Oppanol B12 (polyisobutene)]	porous wafer	0.283 ± 0.632	87.1 ± 12.3 (6)	6.85 ± 1.44	2.93 ± 0.32

a Material information collected from product safety data sheets.

b Surface structure interpretation from electron scanning microscopy—see the Supporting Information.

c Thickness measured via a standard gauge micrometer.

d Vapor permeability (g m^2^ h^–1^) measured through material substrate (see Supporting Information, [*n* = 16]).

**Figure 1:**
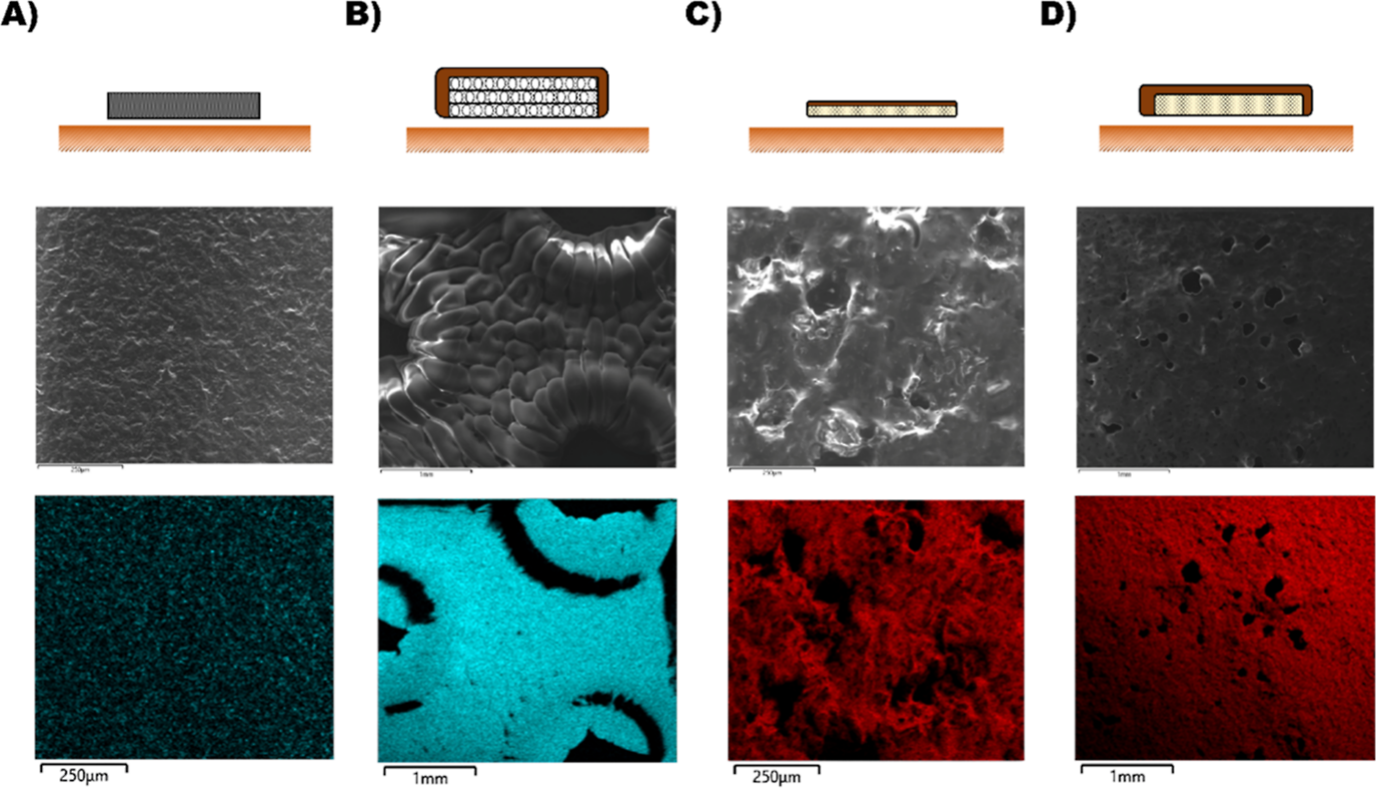
Wound dressing composition. Depiction of overall adhesive structure (top), adhesive surface comparison via scanning electron (middle), and surface elemental distribution analysis (bottom) microscopy of adhesive surfaces of (A) Sil2, (B) Mepilex, (C) Salts hydrocolloid, and (D) Eurotek hydrocolloid. Silica distribution is shown in blue, and carbon distribution is shown in red.

Sample material surfaces were analyzed via Fourier transform infrared (FTIR), Raman spectroscopy, and elemental analysis via SEM—energy-dispersive X-ray (EDX) spectroscopy, and the surface structure of the medical dressings is depicted in Figure 1 and Table 3. The majority of existing dressings contained a porous/perforated structure adhesive reenforced with a strong backing layer, with the hydrocolloids presenting a high carbon (organic) material to the skin, while the silicones presented a solid, continuous sheet with a much lower carbon threshold but a higher % of oxygen. When studied via elemental distribution mapping, silica within the novel silicone exists within this material in a particulate style distribution—indicating that there are plenty of channels for moisture to penetrate the dressing around the hydrophobic silica distribution. Comparatively, the Mepilex, the only other adhesive silica product, has almost no carbon content to separate the silicone material and as such achieves its low vapor transmissions via perforation throughout the material.

**Table 3:** Elemental Composition of Adhesive Materials from SEM–EDX (*n* = 3)

product	C	O	Si	Na
Sil2	52.9 ± 22.2	43.4 ± 20.1	6.8 ± 6.19	
Mepilex		46.1 ± 2.1	53.9 ± 1.9	
Eurotek	27.1 ± 1.4	73.0 ± 1.4		0.5 ± 0.7
Salts	90.6 ± 2.5	8.3 ± 2.5		1.0 ± 0.2

Mechanical testing of the adhesive materials was also carried out to complement their chemical properties. The uniaxial mechanical properties of the adhesive components (isolated from any reinforced backing layer) are described in Table 4 (with raw data presented in the Supporting Information, see Section S9).

**Table 4:** Mechanical Performance of Medical Skin Adhesives Performed by an Instron 5568

product	yield stress (MPa)	stress at break (MPa)	elastic modulus (MPa)	strain at break (%)
Sil2		0.71 ± 0.07	0.33 ± 0.07	803 ± 67
Mepilex^a^				
Eurotek (whole dressing)	0.20 ± 0.01	0.25 ± 0.02	1.37 ± 0.06	472 ± 29
Eurotek (adhesive layer alone)		0.15 ± 0.01	1.36 ± 0.12	98 ± 5
Salts		6.09 ± 0.30	3.34 ± 0.19	610 ± 21

a The Mepilex has a three-layer structure composed of a microporous/uniform polyester thermoset film and perforated adhesive silicones. Because of the layered and perforated structure, mechanical tests produced unreliable measurements, where reliable stress/strain results that represent physical behavior could not be determined.

Here, it was found that the highest mechanical performance was exhibited by the Salts hydrocolloid sample, with the highest elastic modulus (>3 MPa). This, however, would greatly affect its creep performance as a more rigid material is less prone to creep. Similarly, the Eurotek has a high modulus, where the stress–strain curve emerges from the binary structure of the material. It was found that after the initial yield, the hydrocolloid layer underwent immediate mechanical failure, while the fabric backing strain hardens upon breakage of the sample. For this reason, the Eurotek sample was reanalyzed with a single focus on the hydrocolloid adhesive component with the fabric backing removed. This revealed very poor mechanical properties (stress at break of 0.15 MPa) but still a value of elastic modulus which was more than 4 times higher than the novel silicone, in good accordance with the results presented below.

**Figure 2:**
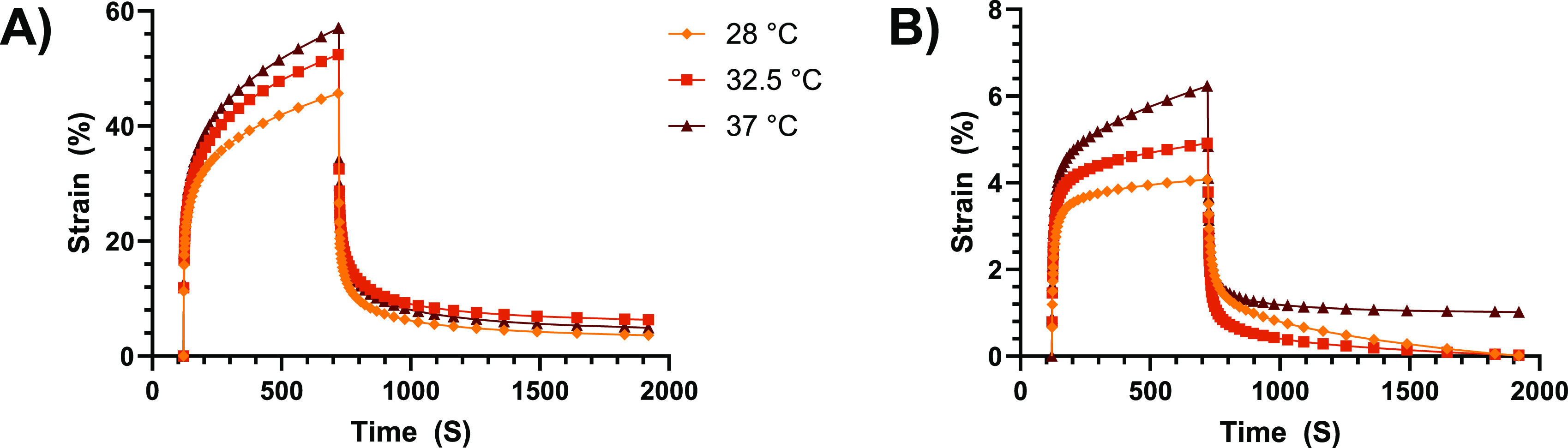
(A) DMA tensile creep test of Sil2 at 28, 32.5, and 37 °C. (B) DMA tensile creep test of the Eurotek hydrocolloid layer at 28, 32.5, and 37 °C.

To understand the viscoelastic impact of these materials on the skin layer, we were interested in measuring the creep response at body relevant temperatures, so DMA and shear rheology tests were carried out to characterize the compounds under tensile and shear stress fields, respectively. As shown in Figure 2, this study was carried out at three varying temperatures between 28 and 37 °C to indicate the potential variations between room and body temperature that skin-adhesive materials will be exposed to.

The tensile tests showed that the modified silicone exhibited greater maximum strain (from 46% at 28 °C to 57% at 37 °C) than is observed in the Eurotek hydrocolloid product. After recovery, the lowest residual strain was recorded at 28 °C (ca. 3.6%), which is not unexpected as at lower temperature, the materials would behave more elastically. The maximum strain potential likely varies because the hydrocolloid products are separated by the fabric layer prior to testing for fair comparison. These specimens exhibited a much lower maximum strain under the same tensile stress (ca. 10-fold lower than Sil2). This indicates that the Sil2 dressing is much more compliant with creep, which might lead to less mechanical stress to be transferred to the skin over time.

**Figure 3:**
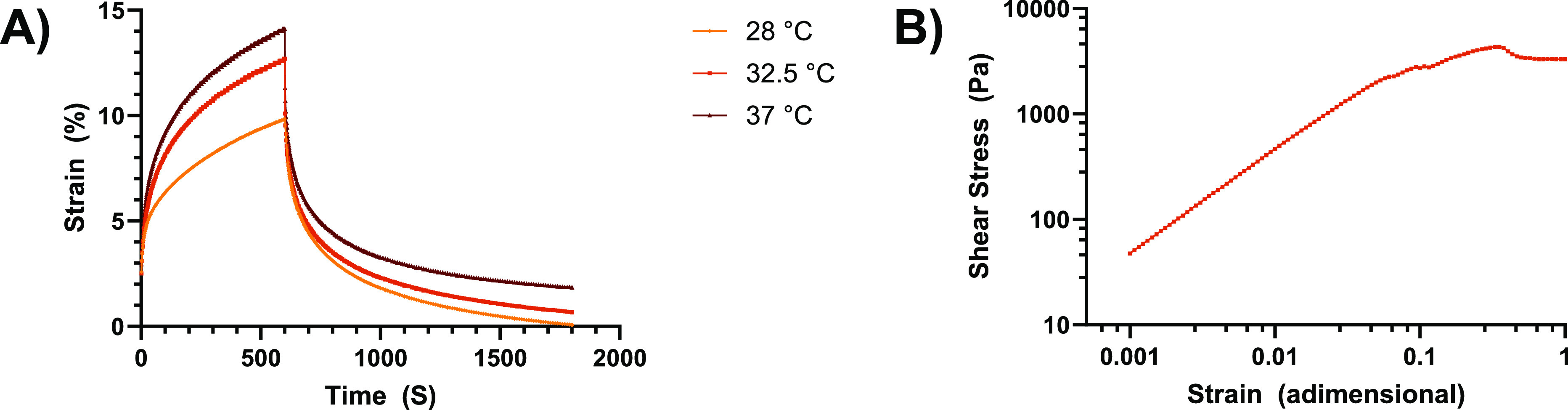
(A) Parallel plate shear creep test of Sil2 at 28, 32.5, and 37 °C. (B) Parallel plate amplitude sweep of Sil2 at 32.5 °C.

As stated earlier, other dressings examined were provided as multilayer and/or perforated structures, making a fair comparison with the novel silicone not possible. The additional presence of many macroscopic pores in the Mepilex reduces dramatically the significance of stress and strain readings, and the Salts products are provided in a format where the shape and size of the sample would not allow the achievement of viable specimens for rheological characterization. The Eurotek hydrocolloid layer presented a dry surface with a very low friction coefficient, exposed from the removal of the polymer fabric side. This did not allow for meaningful parallel plate tests as it would require a significant normal force applied to the specimen during the test, which would greatly affect the results. Therefore, as the only product constituted of a homogeneous material whose properties are not dependent on fabrication of layered individual substrates, it was only possible to continue analysis of the silicone product by parallel plate and amplitude sweep testing, as shown in Figure 3.

The parallel plate tests revealed a similar variation of mechanical properties with the temperature under shear conditions: the maximum strain jumped from ca. 10% at 28 °C to ca. 14% at 37 °C, where the residual shear strain after recovery was also greater (1.9%). The difference with the tensile test results, in terms of extent of creep, indicates highly anisotropic mechanical characteristics. The amplitude sweep test carried out on Sil2 (32.5 °C was chosen as the average skin temperature) confirmed the above considerations: a strongly nonlinear viscoelastic response was exhibited by the silicone compound for shear strains greater than 5%, demonstrating that the material exhibits a rather narrow linear viscoelastic region.

**Figure 4:**
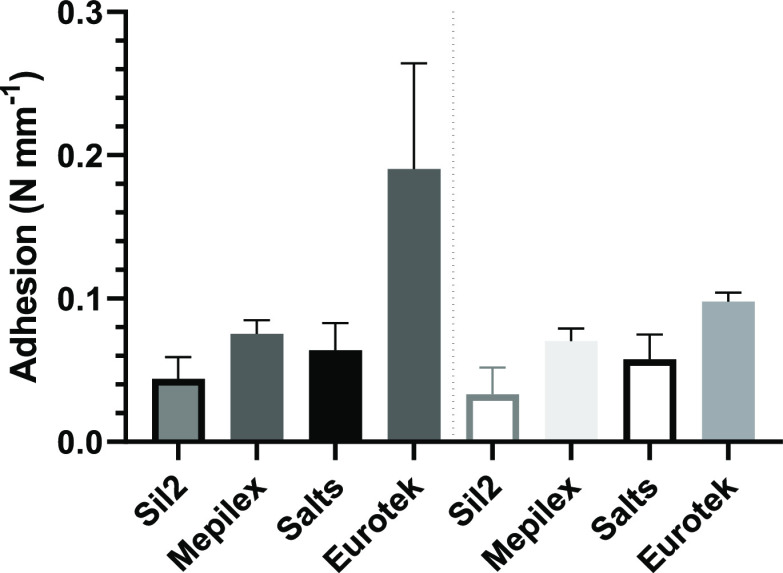
Peak peel (left) and steady-state (right) adhesion between Sil2 (*n* = 6), Mepilex (*n* = 7), Salts hydrocolloid (*n* = 7), and Eurotek hydrocolloid (*n* = 7/5) measured against participant forearm skin.

A small number of participants (*N* = 7) undertook additional peel strength tests where a long strip of each adhesive dressing was gently removed from the participants’ forearm, while the resistive force was measured (Figure 4). During these measurements, irregularities in the data of one of the silicone dressings resulted in that data set being discounted, and only 50% of measurements of the thicker Eurotek hydrocolloid achieved a useable steady-state peel strength (raw data are presented in Section S6).

The novel silicone composite had a peak adhesion strength of 0.044 ± 0.035 N mm^–1^, which is the lowest of those of the adhesive materials tested. The next nearest was the Salts HC, Mepilex-perforated silicone, and Eurotek HC (0.0064 ± 0.017, 0.075 ± 0.009, and 0.190 ± 0.074 N mm^–1^, respectively). When the samples were analyzed to compare the existing adhesive dressings to the novel silicone, the Cohen’s *d* effect size was found to be 0.37 for the Salts–indicating a medium effect and 0.53 and 1.25 for the Mepilex and Eurotek adhesives, respectively, indicating a large effect size. Similar data trends were found for the steady-state adhesion which was found to be 0.033 ± 0.019, 0.058 ± 0.017, 0.070 ± 0.009, and 0.098 ± 0.006 N mm^–1^ respectively, with all three prior adhesive materials indicating a Cohen’s *d* effect size >0.5 when compared to the novel silicone composite.

At this point, as the Salts HC exhibited the lowest peel strength and highest TEWL of the HC dressings, it was selected to be compared to the novel silicone products in a larger participant skin contact trial. A comparative biocompatibility of these two materials was carried out to show equivalent noncytotoxicity against human fibroblast cells (F45) of these two materials (see Supporting Information, Section S3). The comparison of the adhesive properties of biocompatible hydrophobic dressings was carried out using a participant study of either 6 or 24 h in duration. Across the participant study, a small number of patches detached early during the participant wear time. These data points were noted, but the data were discarded for the purposes of analysis (see Section S5).

TEWL is an inherently variable skin property, and so a control measurement on 1M and 1F volunteers across a 3 week period provides a baseline for skin properties (see Section S7). These showed that there was no significance to the date/time that the measurements were taken, but there were substantial differences between the forearms and torso measurements—results that indicate a greater degree of variation than that was observed in our review of literature.

**Figure 5:**
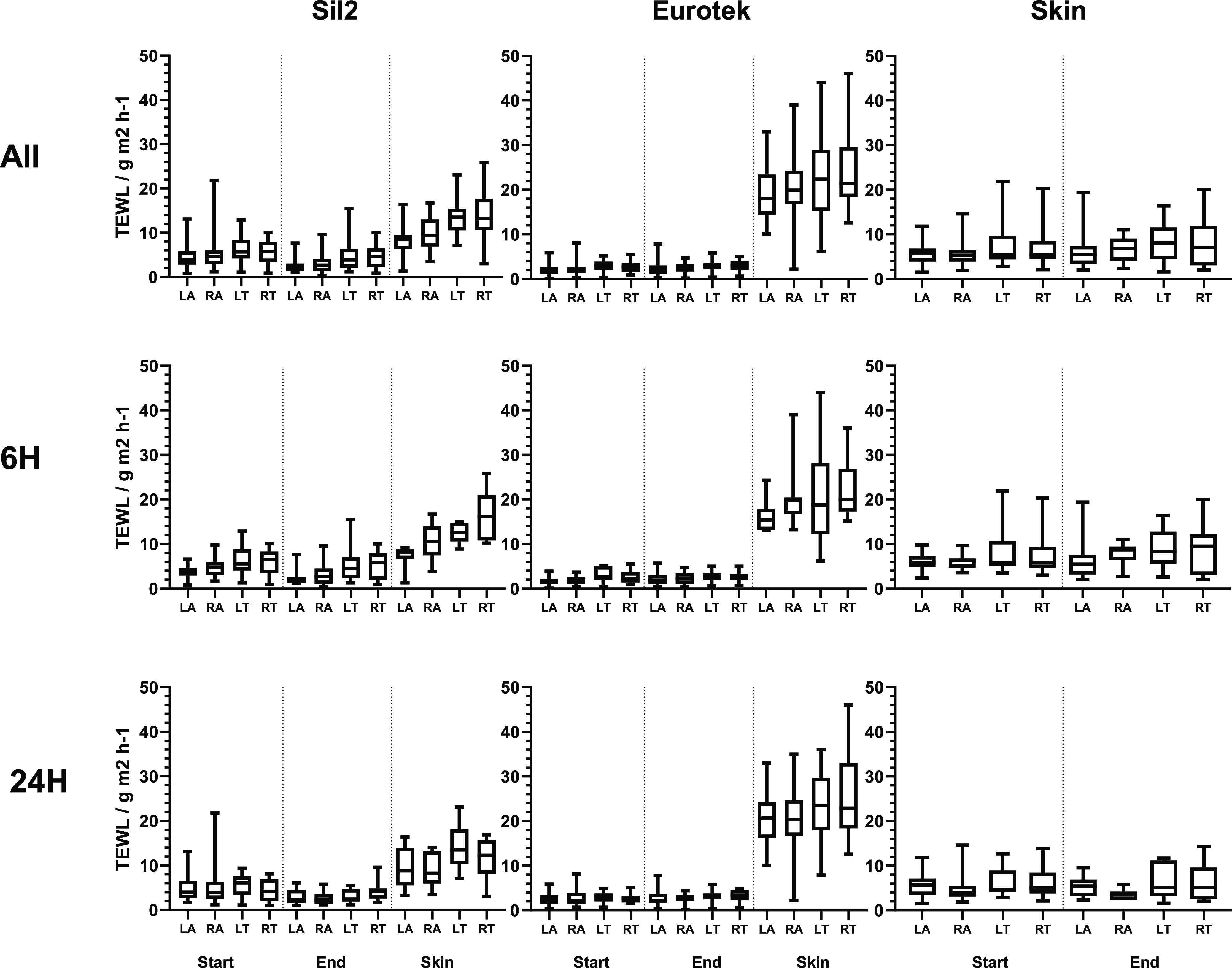
Box and Whisker summary of TEWL data of participant trial showing data for all participants (top row, *n* = 31), or just 6 h (middle row, *n* = 18) or 24 h (bottom row, *n* = 13). Each graph contains four sets of data each at onset of wear after dressing is applied, at end of wear before the patch is removed, and skin measurement after patch removal. Data is presented in groups of left arm (LA), right arm (RA), left torso (LT), and right torso (RT). Graphs show data for silicone patches (left column), hydrocolloid patches (middle column), and control group of skin (right column).

The data from the TEWL analysis of participants wearing skin adhesives are shown in Figure 5. Comparisons between TEWL measurements at 6 and 24 h were carried out using a two-way analysis of variance (ANOVA), and it was found that there was no significant difference between the TEWL of participants who wore the dressings for different time periods (*P* = 0.3092, see Section S4). Two-way ANOVA comparisons also showed that there was no significant difference between the TEWL of left and right arms (*P* = 0.08) or left and right torso (*P* = 0.56)—however, there was a significant difference between arms and torso (*P* < 0.001), indicating that the skin and dressings have different behaviors across the two body regions.

The data showed that at the onset of the study, when dressings are applied to the skin (which had a TEWL of 6.5 ± 0.96), the silicone dressing had a TEWL significantly higher than the hydrocolloid (silicone TEWL 5.21 ± 0.76 and hydrocolloid TEWL 2.52 ± 0.41). The difference between the hydrocolloid and the silicone adhesives is significantly different as shown by an unpaired *t*-test (*P* < 0.0010), and whist the silicone dressing TEWL is closer to skin, the difference is still statistically significant (*P* < 0.01).

As the study progressed, there is no statistical significance between the TEWL of the hydrocolloid dressing at the start and end of the study (ANOVA *P* = 0.3542) or the control skin (ANOVA *P* = 0.1793). The silicone dressing, however, showed varying behavior depending on whether the arm or torso patch was tested. In both, a slight decrease in TEWL is observed for both the 6 and 24 h participant (no statistical difference is seen between the two data sets, two-way ANOVA *P* = 0.14), with the arms showing a decrease in TEWL (2.81 ± 1.87), while the torso shows a slight but substantially reduced decrease in TEWL to reach an average of 4.70 ± 3.11.

**Figure 6:**
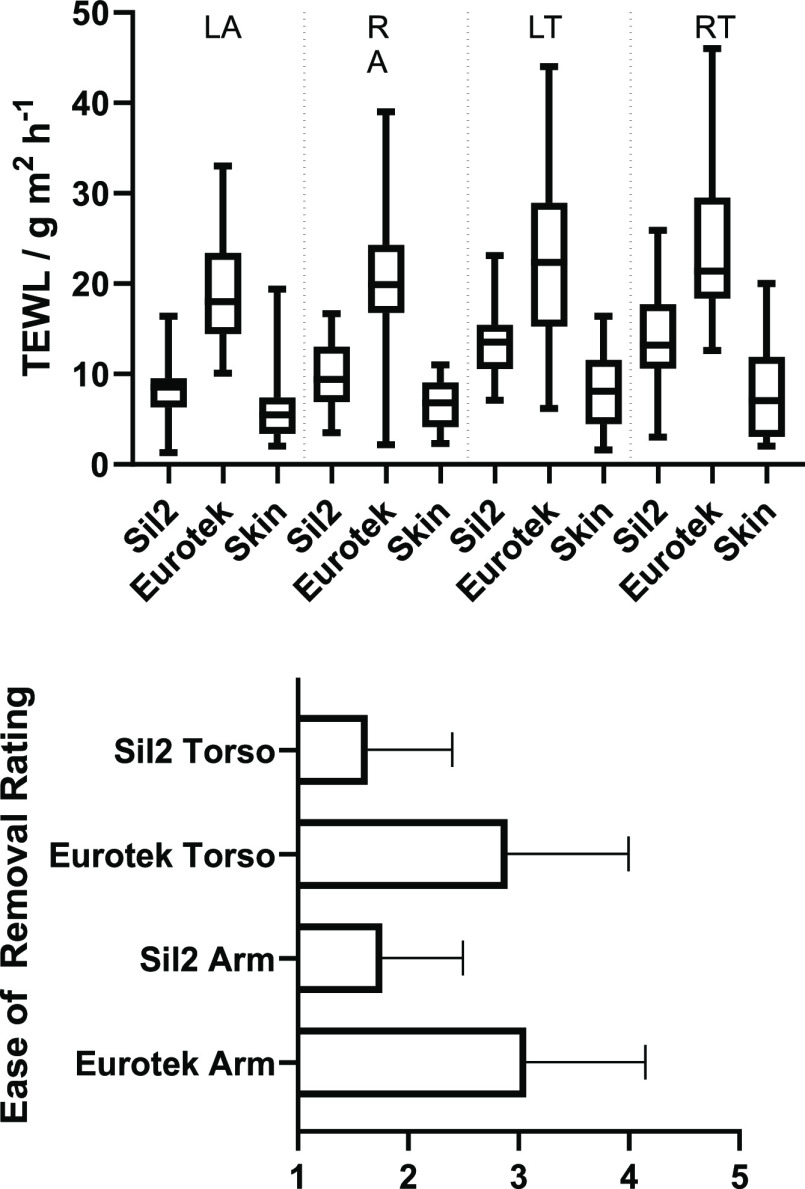
Top: TEWL of the skin beneath adhesive dressings of silicone, hydrocolloid, and control skin measurement of the left arm (LA), right arm (RA), left torso (LT), and right torso (RT). Bottom: participant response to question about ease of removal.

The most significant observation from the analysis of TEWL, however, was seen in the analysis of skin beneath the hydrocolloid and silicone patches, as measured after the dressings were removed (Figure 6). The TEWL of the skin beneath the hydrocolloid dressing showed a small increase compared to the control skin, rising to 11.3 ± 4.89, which when compared via a *t*-test is a statistically significant difference (*P* < 0.0001), but the skin beneath the hydrocolloid dressing had increased to reach an average of 21.6 ± 8.01.

**Figure 7:**
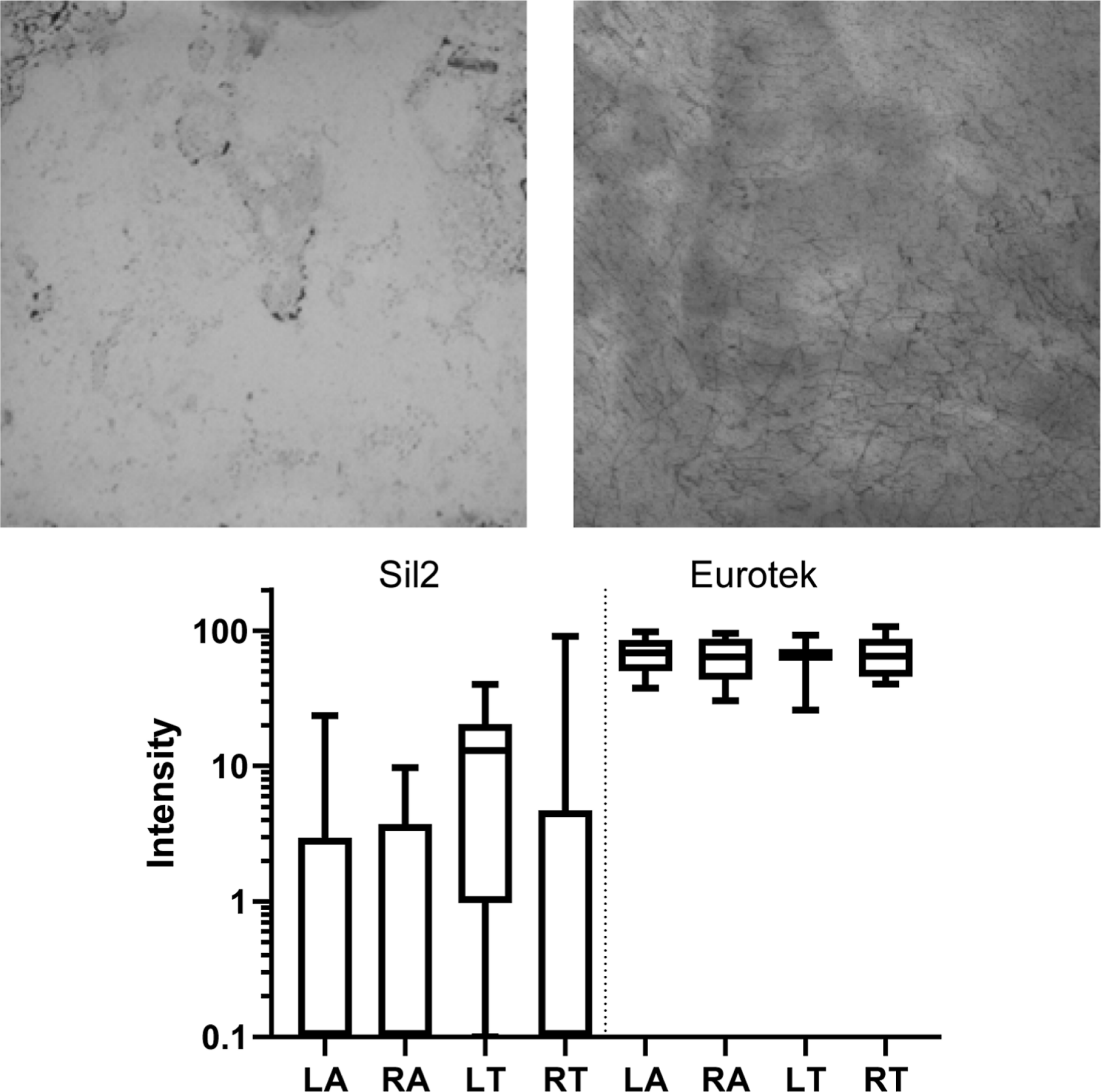
Top: typical stained protein images were measured under a light microscope to compare protein staining of Sil2 (left) and Eurotek (right) dressings compared to background controls. Bottom: mean intensity of protein adhered to the hydrocolloid and silicon dressings.

All participants (whole study) were also asked to comment upon the ease of removal (pain/discomfort levels) of the patches in both arms and torso, and a significant proportion indicated that they felt less pain/discomfort in the removal of the silicone dressing. Participants were asked to comment on the ease of removal (a pain rating) of the dressings on a scale of 1–5 (5 being the most painful and 1 being the least). Participants were unanimous in their response that the silicon dressing was easier to remove; however, the subjective nature of the question led to large variety between the responses, as shown in Figure 7. The average ease of removal for silicones was 1.7 ± 0.7, while the ease of removal for hydrocolloids was 3.0 ± 1.0. Almost no participants reported a difference in the removal between the torso and the arms.

The dressings removed from the participants were taken for counterstaining using colloidal Coomassie dye, as shown in Figure 7. The average increase in stain intensity on the silicone dressing compared to the control strip was 7.2 ± 16.4, while that of the hydrocolloid dressings increased by 66.1 ± 19.9. This meant that there was a significant difference between the amount of protein adhered to the two dressings (*P* < 0.001); however, there was no significant difference between the arms or torso of any individual set, (*t*-test *P* > 0.05). Full detailed analysis and comparisons (such as male vs female results) are shown in the Supporting Information (see Section S8).

**Figure 8:**
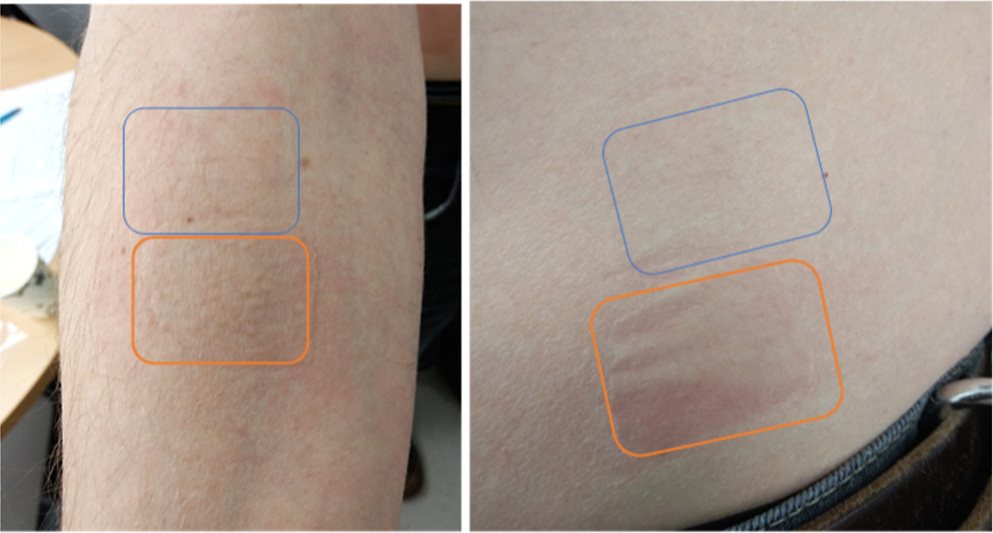
Left: image shows the right arm of a male participant. Blue box represents the area of skin where the silicone dressing was applied and removed from. Orange box represents where the hydrocolloid was applied and removed from. Right: the left torso of the same participant. Orange box represents where hydrocolloid was removed, and the blue box represents where silicone was removed from.

There was no distinction between the protein stripping potential of the male and female participants in any body site, as shown in the Supporting Information. Although visually a slight discrepancy can be observed between the stripping potential of the silicones in the arms and torso, with the torso demonstrating increased range (maximum intensity 91 compared to 23), there is no statistical significance in the protein removal as the standard deviation for both are within each other’s interquartile range. A comparison of the data via *t*-test showed a *P* > 0.05. Skin damage of the participants was assessed by taking photographs of any site where reddening was observed. Figure 8 shows an image of an extreme case where they exhibited severe reddening following wear of the hydrocolloid for just 6 h.

**Figure 9:**
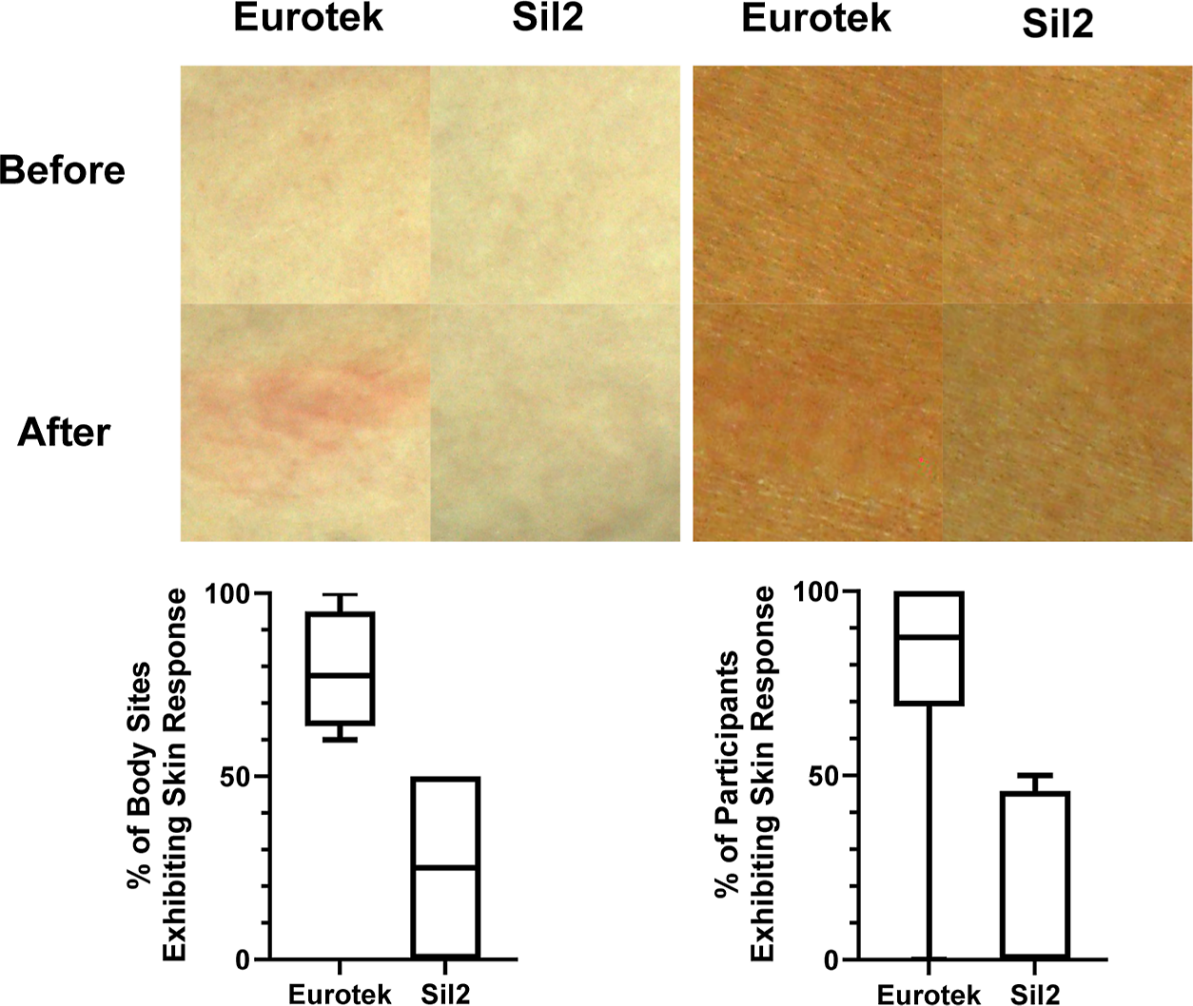
Top left: comparison of skin before and after patches were applied showed erythema. Top right: comparisons of skin before and after patches were applied showed skin stripping (detexturization). Bottom left: box and Whisker plots show interquartile ranges of skin response when grouped by body sites. Bottom right: Box and whisker plots showing interquartile ranges of skin response when grouped by participants.

A selection of 10 participants was then analyzed using a 3D-polarized light camera so the skin surface could be analyzed after the study was completed (Figure 9). This allowed us to create a three-dimensional representation of the participant test site pre, during, and post patch wearing to allow a digital post examination of skin response to patch wearing.

Skin sites pre and post patch wearing were then compared and designated as responsive (photographs showed some form of skin damage, i.e., reddening, erythema, flattening, or peeling) or nonresponsive to the patches. This provided an indication of what % of skin sites showed a response but not the degree or severity of the skin damage. However, when grouped by body site (i.e., three out of four left arm patches showed a skin response), 70% of hydrocolloid patches showed signs of skin damage, while only 17% of silicone samples showed negative skin response. This data is summarized with examples in Figure 9 and further expanded on in the Supporting Information.

## Discussion and Conclusions

The hydrophobically modified novel silicone shows a higher vapor permeability potential than many available medical hydrocolloid or silicone adhesive dressings and utilizes a very different system of moisture management, as shown by the humidity tests. Even though neither material could be described as hydrophilic, both are designed to contain elements that control the passage of moisture from the skin—one via swelling and hydration and the other via passive diffusion. It is therefore not surprising to see that in higher levels of moisture, when the hydrocolloid material is swollen, there is a lower vapor permeability—indicating greater potential for these materials to occlude the skin. This is borne out in the participant study, which shows a reduced TEWL for the hydrocolloid adhesive both at the start and at the end of the study compared to the silicone dressing. As a result of this phenomenon, when the patch is removed, participants demonstrate increased levels of skin irritancy and a potentially associated increase in TEWL. Skin maceration is a known phenomenon to follow increased hydration levels, and we have observed significant increases in both TEWL and erythema in the skin beneath the hydrocolloid patches within just a few hours of wear that were not present beneath the Sil2 patches.

**Figure 10:**
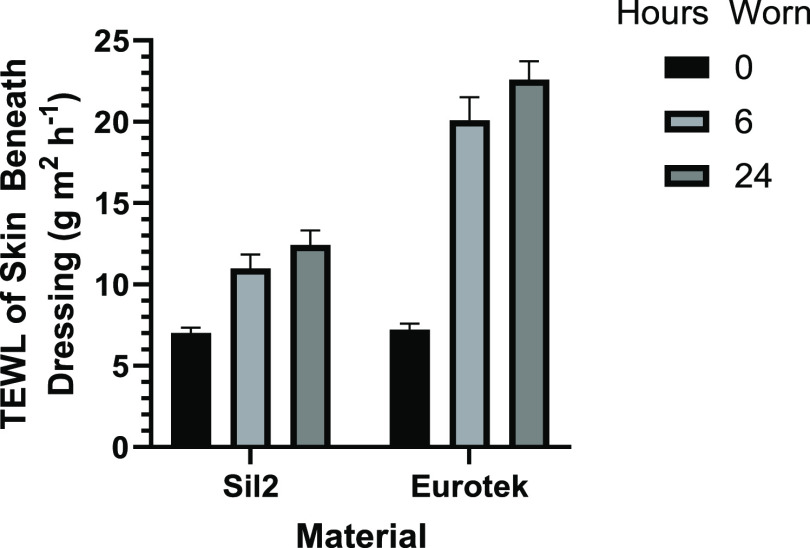
Increase in skin TEWL beneath dressing over 6 and 24 h wear; error bars show standard error.

The reduced peel strength of the Sil2 product results in significantly less-protein stripping potential than the hydrocolloid tested. Studies on microdermabrasion show that it takes between 12 and 24 h for the stratum corneum to regenerate after the upper layer of corneocytes has been damaged.^56^ As a result, post patch removal, the skin will have reduced barrier properties until it has regenerated. Reducing the volume of proteins stripped could also have an impact on the observed erythema, respectively. There was no statistical difference in the number of participants who observed irritation from the hydrocolloid dressings by gender or by the time that they wore the patch (6 h compared to 24 h). This is interesting as there was a significant difference in the observed skin properties—indicating a progression of increasing TEWL with increased wear time, as shown in Figure 10, although further testing with an increased number of participants and time points may be required to fully disclose the significance of this data. What can be observed though is that during 24 h of wear, the silicone increased the skin TEWL by an average of 80%, while the hydrocolloid increased it by approximately 300% over an equivalent period.

The viscoelastic behavior and creep response of the novel silicone provide further evidence that less stress is likely to be transferred to the skin over time by the silicone compounds. The high creep compliance united with the anisotropic and nonlinear mechanical properties of the novel silicone is likely to be a result of its peculiar composite morphology.

Further work will likely need to be undertaken to fully disassociate the interactions between the modified silicone product and participant skin, but in all metrics tested (decreased skin-stripping peel force, decreased protein removal from the stratum corneum, decreased skin occlusion, decreased skin irritancy, and decreased participant pain response), it demonstrates a marked improvement in comfort and wear over previous technologies.

## Supporting Information Available

adhesive dressing microscopic analysis, moisture management comparisons, staining of human fibroblast cells, TEWL ANOVA data, patch loss rate, peel adhesion tests, TEWL variability, stain analysis, and uniaxial mechanical testing raw data (PDF)

## Abbreviations

HC: hydrocolloid
RH: room humidity
SC: stratum corneum
TEWL: trans-epidermal water loss
VP: vapor permeability

## References

[ref1] Cutting K. F. Impact of Adhesive Surgical Tape and Wound Dressings on the Skin, with Reference to Skin Stripping. J. Wound Care 2008, 17 (4), 157–162. 10.12968/jowc.2008.17.4.28836. 18494433

[ref2] Kruse C. R.; Nuutila K.; Lee C. C. Y.; Kiwanuka E.; Singh M.; Caterson E. J.; Eriksson E.; Sørensen J. A. The External Microenvironment of Healing Skin Wounds. Wound Repair Regen. 2015, 23 (4), 456–464. 10.1111/wrr.12303. 25857996

[ref3] Rippon M.; Ousey K.; Rogers A.; Atkin L. Wound Hydration versus Maceration: Understanding the Differences. Wounds 2016, 12 (3), 62–68.

[ref4] Chowdhry M.; Chen A. F. Wound Dressings for Primary and Revision Total Joint Arthroplasty. Ann. Transl. Med. 2015, 3 (18), 268 10.3978/J.ISSN.2305-5839.2015.09.25. 26605314 PMC4630542

[ref5] Vowden K.; Vowden P. Wound Dressings: Principles and Practice. Surgery 2017, 35 (9), 489–494. 10.1016/j.mpsur.2017.06.005.

[ref6] Pang C.; Ibrahim A.; Bulstrode N. W.; Ferretti P. An Overview of the Therapeutic Potential of Regenerative Medicine in Cutaneous Wound Healing. Int. Wound J. 2017, 14 (3), 450–459. 10.1111/iwj.12735. 28261962 PMC7949790

[ref7] Han G.; Ceilley R. Chronic Wound Healing: A Review of Current Management and Treatments. Adv. Ther. 2017, 34 (3), 599–610. 10.1007/s12325-017-0478-y. 28108895 PMC5350204

[ref8] Schäfer P.; Bewick-Sonntag C.; Capri M. G.; Berardesca E. Physiological Changes in Skin Barrier Function in Relation to Occlusion Level, Exposure Time and Climatic Conditions. Skin Pharmacol. Physiol. 2002, 15 (1), 7–19. 10.1159/000049384. 11803253

[ref9] Fartasch M.; Taeger D.; Broding H. C.; Schöneweis S.; Gellert B.; Pohrt U.; Brüning T. Evidence of Increased Skin Irritation after Wet Work: Impact of Water Exposure and Occlusion. Contact Dermatitis 2012, 67 (4), 217–228. 10.1111/j.1600-0536.2012.02063.x. 22591550

[ref10] Alvey B.; Beck D. E. Peristomal Dermatology. Clin. Colon Rectal Surg. 2008, 21 (1), 041–044. 10.1055/s-2008-1055320. PMC2780183 20011395

[ref11] Chen K.; Ren J.; Chen C.; Xu W.; Zhang S. Safety and Effectiveness Evaluation of Flexible Electronic Materials for next Generation Wearable and Implantable Medical Devices. Nano Today 2020, 35, 100939 10.1016/j.nantod.2020.100939.

[ref12] Yu B.; Kang S. Y.; Akthakul A.; Ramadurai N.; Pilkenton M.; Patel A.; Nashat A.; Anderson D. G.; Sakamoto F. H.; Gilchrest B. A.; Anderson R. R.; Langer R. An Elastic Second Skin. Nat. Mater. 2016, 15 (8), 911–918. 10.1038/nmat4635. 27159017

[ref13] Hansen D.; Zajforoushan Moghaddam S.; Eiler J.; Hansen K.; Thormann E. Performance of Polymeric Skin Adhesives during Perspiration. ACS Appl. Polym. Mater. 2020, 2 (4), 1535–1542. 10.1021/acsapm.9b01214.

[ref14] Lager P.; Loxdale L. Use of Breathable Silicone Technology in an Ostomy Appliance Flange. Br. J. Nurs. 2021, 30 (Sup8), 25–35. 10.12968/bjon.2021.30.Sup8.25. 34106774

[ref15] Swift T. Peristomal Skin Complications: New Materials Needed to Ease the Ostomy Care Market. Br. J. Dermatol. 2023, 188 (4), 455–456. 10.1093/bjd/ljad006. 36763801

[ref16] Pearce L.; Lee S. Skin Compatible Silicone Composition. U.S. Patent 20,210,346,569 A1, 2021.

[ref17] Swift T.; Westgate G.; Van Onselen J.; Lee S. Developments in Silicone Technology for Use in Stoma Care. Br. J. Nurs. 2020, 29 (6), S6–S15. 10.12968/bjon.2020.29.6.S6. 32207652

[ref18] Belfield-Cockings K. A New Approach with a Novel Silicone Adhesive Stoma Bag: A Clinical Study on Peristomal Skin and Quality of Life. Br. J. Nurs. 2022, 31 (6), S40–S47. 10.12968/bjon.2022.31.6.S40. 35333552

[ref19] Salih S. I.; Oleiwi J. K.; Ali H. M. Study the Mechanical Properties of Polymeric Blends (SR/PMMA) Using for Maxillofacial Prosthesis Application. Int. Conf. Mater. Eng. Sci. 2018, 454, 012086 10.1088/1757-899X/454/1/012086.

[ref20] Matsui T.; Amagai M. Dissecting the Formation, Structure and Barrier Function of the Stratum Corneum. Int. Immunol. 2015, 27 (6), 269–280. 10.1093/intimm/dxv013. 25813515

[ref21] Machado M.; Salgado T. M.; Hadgraft J.; Lane M. E. The Relationship between Transepidermal Water Loss and Skin Permeability. Int. J. Pharm. 2010, 384 (1–2), 73–77. 10.1016/j.ijpharm.2009.09.044. 19799976

[ref22] Elias P. M. Skin Barrier Function. Curr. Allergy Asthma Rep. 2008, 8 (4), 299–305. 10.1007/s11882-008-0048-0. 18606081 PMC2843412

[ref23] Taylor N. A.; Machado-Moreira C. A. Regional Variations in Transepidermal Water Loss, Eccrine Sweat Gland Density, Sweat Secretion Rates and Electrolyte Composition in Resting and Exercising Humans. Extreme Physiol. Med. 2013, 2 (1), 4 10.1186/2046-7648-2-4. PMC3710196 23849497

[ref24] Gioia F.; Celleno L. The Dynamics of Transepidermal Water Loss (TEWL) from Hydrated Skin. Skin Res. Technol. 2002, 8 (3), 178–186. 10.1034/j.1600-0846.2002.10342.x. 12236888

[ref25] Busch K.-H.; Aliu A.; Walezko N.; Aust M. Medical Needling: Effect on Moisture and Transepidermal Water Loss of Mature Hypertrophic Burn Scars. Cureus 2018, 10 (3), 2365 10.7759/cureus.2365. PMC5969801 29805934

[ref26] Gao Y.; Wang X.; Chen S.; Li S.; Liu X. Acute Skin Barrier Disruption with Repeated Tape Stripping: Anin Vivomodel for Damage Skin Barrier. Skin Res. Technol. 2013, 19 (2), 162–168. 10.1111/srt.12028. 23279155

[ref27] Farris M. K.; Petty M.; Hamilton J.; Walters S. A.; Flynn M. A. Medical Adhesive-Related Skin Injury Prevalence among Adult Acute Care Patients a Single-Center Observational Study. J. Wound, Ostomy Cont. Nurs. 2015, 42 (6), 589–598. 10.1097/WON.0000000000000179. 26528871

[ref28] Gray M.; Black J. M.; Baharestani M. M.; Bliss D. Z.; Colwell J. C.; Goldberg M.; Kennedy-Evans K. L.; Logan S.; Ratliff C. R. Moisture-Associated Skin Damage: Overview and Pathophysiology. J. Wound, Ostomy Cont. Nurs. 2011, 38 (3), 233–241. 10.1097/WON.0b013e318215f798. 21490547

[ref29] Seaman S. Dressing Selection in Chronic Wound Management. J. Am. Podiatr. Med. Assoc. 2002, 92 (1), 24–33. 10.7547/87507315-92-1-24. 11796796

[ref30] Cronin E. Silicone-Based Stoma Accessories in Clinical Practice. Br. J. Nurs. 2016, 25 (5), 28–34. 10.12968/bjon.2016.25.5.s28. 26973010

[ref31] Mojsiewicz-Pieńkowska K.; Jamrógiewicz M.; Żebrowska M.; Mikolaszek B.; Sznitowska M. Double Layer Adhesive Silicone Dressing as a Potential Dermal Drug Delivery Film in Scar Treatment. Int. J. Pharm. 2015, 481 (1–2), 18–26. 10.1016/j.ijpharm.2015.01.050. 25639195

[ref32] Zillmer R.; Agren M. S.; Gottrup F.; Karlsmark T. Biophysical Effects of Repetitive Removal of Adhesive Dressings on Peri-Ulcer Skin. J. Wound Care 2006, 15 (5), 187–191. 10.12968/jowc.2006.15.5.26907. 16711170

[ref33] Stewart S. A.; Dougall G. M. G.; Tafuro E. M. The Use of Silgel STC-SE, a Topical Silicone Gel for the Treatment and Reduction of Hypertrophic and Keloid Scars. Plast. Reconstr. Surg.—Glob. Open 2016, 4 (12), 1183 10.1097/GOX.0000000000001183. PMC5222672 28293527

[ref34] Marshall C. D.; Hu M. S.; Leavitt T.; Barnes L. A.; Lorenz H. P.; Longaker M. T. Cutaneous Scarring: Basic Science, Current Treatments, and Future Directions. Adv. Wound Care 2018, 7 (2), 29–45. 10.1089/wound.2016.0696. PMC5792238 29392092

[ref35] Meaume S.; Le Pillouer-Prost A.; Richert B.; Roseeuw D.; Vadoud J. Management of Scars: Updated Practical Guidelines and Use of Silicones. Eur. J. Dermatol. 2014, 24 (4), 435–443. 10.1684/ejd.2014.2356. 25141160

[ref36] Hadgraft J.; Lane M. E. Transepidermal Water Loss and Skin Site: A Hypothesis. Int. J. Pharm. 2009, 373 (1–2), 1–3. 10.1016/j.ijpharm.2009.02.007. 19429281

[ref37] Bouwstra J. A.; De Graaff A.; Gooris G. S.; Nijsse J.; Wiechers J. W.; Van Aelst A. C. Water Distribution and Related Morphology in Human Stratum Corneum at Different Hydration Levels. J. Invest. Dermatol. 2003, 120 (5), 750–758. 10.1046/j.1523-1747.2003.12128.x. 12713576

[ref38] Warner R. R.; Stone K. J.; Boissy Y. L. Hydration Disrupts Human Stratum Corneum Ultrastructure. J. Invest. Dermatol. 2003, 120 (2), 275–284. 10.1046/j.1523-1747.2003.12046.x. 12542533

[ref39] Whitehead F.; Giampieri S.; Graham T.; Grocott P. Identifying, Managing and Preventing Skin Maceration: A Rapid Review of the Clinical Evidence. J. Wound Care 2017, 26 (4), 159–165. 10.12968/jowc.2017.26.4.159. 28379098

[ref40] Buraczewska I.; Broström U.; Lodén M. Artificial Reduction in Transepidermal Water Loss Improves Skin Barrier Function. Br. J. Dermatol. 2007, 157 (1), 82–86. 10.1111/j.1365-2133.2007.07965.x. 17553058

[ref41] Bock M.; Damer K.; Wulfhorst B.; John S. M. Semipermeable Glove Membranes-Effects on Skin Barrier Repair Following SLS Irritation. Contact Dermatitis 2009, 61 (5), 276–280. 10.1111/j.1600-0536.2009.01622.x. 19878242

[ref42] Jungersted J. M.; Høgh J. K.; Hellgren L. I.; Jemec G. B. E.; Agner T. Skin Barrier Response to Occlusion of Healthy and Irritated Skin: Differences in Trans-Epidermal Water Loss, Erythema and Stratum Corneum Lipids. Contact Dermatitis 2010, 63 (6), 313–319. 10.1111/j.1600-0536.2010.01773.x. 20731690

[ref43] Welzel J.; Wilhelm K. P.; Wolff H. H. Skin Permeability Barrier and Occlusion: No Delay of Repair in Irritated Human Skin. Contact Dermatitis 1996, 35 (3), 163–168. 10.1111/j.1600-0536.1996.tb02335.x. 8930477

[ref44] Hoeksema H.; De Vos M.; Verbelen J.; Pirayesh A.; Monstrey S. Scar Management by Means of Occlusion and Hydration: A Comparative Study of Silicones versus a Hydrating Gel-Cream. Burns 2013, 39 (7), 1437–1448. 10.1016/j.burns.2013.03.025. 23639224

[ref45] Dabboue H.; Builles N.; Frouin E. ´.; Scott D.; Ramos J.; Marti-Mestres G. Assessing the Impact of Mechanical Damage on Full-Thickness Porcine and Human Skin Using AnIn VitroApproach. BioMed Res. Int. 2015, 2015, 1–10. 10.1155/2015/434623. PMC4515495 26247021

[ref46] Klotz T.; Ibrahim A.; Maddern G.; Caplash Y.; Wagstaff M. Devices Measuring Transepidermal Water Loss: A Systematic Review of Measurement Properties. Skin Res. Technol. 2022, 28 (4), 497–539. 10.1111/srt.13159. 35411958 PMC9907714

[ref47] Fluhr J. W.; Feingold K. R.; Elias P. M. Transepidermal Water Loss Reflects Permeability Barrier Status: Validation in Human and Rodent in Vivo and Ex Vivo Models. Exp. Dermatol. 2006, 15 (7), 483–492. 10.1111/j.1600-0625.2006.00437.x. 16761956

[ref48] Grove G. L.; Zerweck C. R.; Houser T. P.; Smith G. E.; Koski N. I. A Randomized and Controlled Comparison of Gentleness of 2 Medical Adhesive Tapes in Healthy Human Subjects. J. Wound, Ostomy Cont. Nurs. 2013, 40 (1), 51–59. 10.1097/won.0b013e318276f2a4. 23202590

[ref49] Luebberding S.; Krueger N.; Kerscher M. Age-Related Changes in Skin Barrier Function - Quantitative Evaluation of 150 Female Subjects. Int. J. Cosmet. Sci. 2013, 35 (2), 183–190. 10.1111/ics.12024. 23113564

[ref50] Luebberding S.; Krueger N.; Kerscher M. Skin Physiology in Men and Women:In Vivoevaluation of 300 People Including TEWL, SC Hydration, Sebum Content and Skin Surface PH. Int. J. Cosmet. Sci. 2013, 35 (5), 477–483. 10.1111/ics.12068. 23713991

[ref51] Döge N.; Avetisyan A.; Hadam S.; Pfannes E. K. B.; Rancan F.; Blume-Peytavi U.; Vogt A. Assessment of Skin Barrier Function and Biochemical Changes of Ex Vivo Human Skin in Response to Physical and Chemical Barrier Disruption. Eur. J. Pharm. Biopharm. 2017, 116, 138–148. 10.1016/j.ejpb.2016.12.012. 28012990

[ref52] Zhang Q.; Murawsky M.; Lacount T.; Kasting G. B.; Li S. K. Transepidermal Water Loss and Skin Conductance as Barrier Integrity Tests. Toxicol. Vitro 2018, 51, 129–135. 10.1016/j.tiv.2018.04.009. PMC6082371 29698667

[ref53] Peer R. P.; Burli A.; Maibach H. I. Unbearable Transepidermal Water Loss (TEWL) Experimental Variability: Why?. Arch. Dermatol. Res. 2022, 314 (2), 99–119. 10.1007/s00403-021-02198-y. 33638033

[ref54] Clausen M.-L.; Slotved H.-C.; Krogfelt K. A.; Agner T. Tape Stripping Technique for Stratum Corneum Protein Analysis. Sci. Rep. 2016, 6, 19918 10.1038/srep19918. 26817661 PMC4730153

[ref55] Goulet-Pelletier J.-C.; Cousineau D. A Review of Effect Sizes and Their Confidence Intervals, Part I: The Cohen’sd Family. Quant. Meth. Psych. 2018, 14 (4), 242–265. 10.20982/tqmp.14.4.p242.

[ref56] Andrews S.; Lee J. W.; Prausnitz M. Recovery of Skin Barrier after Stratum Corneum Removal by Microdermabrasion. AAPS PharmSciTech 2011, 12 (4), 1393–1400. 10.1208/s12249-011-9715-x. 22009306 PMC3225536

