# Supplementary material for: Evaluating the Irritant Factors of Silicone and Hydrocolloid Skin Contact Adhesives Using Trans-Epidermal Water Loss, Protein Stripping, Erythema, and Ease of Removal
Source: ACS Appl Bio Mater. 2023 Dec 27;7(1):284–96. doi: 10.1021/acsabm.3c00874 (PMC10792606; doi:10.1021/acsabm.3c00874)
Supplement: Supplementary file 1 — mt3c00874_si_001.pdf [file mt3c00874_si_001.pdf]

Evaluating the Irritant Factors of Silicone and Hydrocolloid Skin Contact Adhesives using Trans-Epidermal Water Loss, Protein Stripping, Erythema and Ease of Removal

Supporting Information

Edward Dyson,[a] Stephen Sikkink,[a] Davide Nocita,[b] Peter Twigg,[b] Gill Westgate,[a] Thomas Swift[a]*

[a] School of Chemistry and Biosciences, University of Bradford, United Kingdom, BD7 1DP

[b] Faculty of Engineering and Informatics, University of Bradford, United Kingdom, BD7 1DP

* Email address for correspondence: T.Swift@bradford.ac.uk

Contents

1. Adhesive Dressing Microscopy Analysis 2
2. Adhesive Dressing Moisture Management Comparisons 7
3. Staining of Human Fibroblast Cells 10
4. TEWL ANOVA Data 13
5. Patch Loss Rate 14
6. Peel Adhesion Tests 15
7. TEWL Variability 17
8. Stain Analysis 18
9. Uniaxial Mechanical Testing 19

1. Adhesive Dressing Microscopy Analysis

FTIR

FTIR analysis of the materials showed the different functional chemistry of the adhesive surface as shown in **Figure S1**. Assignment of primary peaks in the silicone FTIR spectra was carried out following the work of Salih *et al.*[1]

Silicone: 1080 (stretching vibration Si-O-Si), 1250 (bending vibration Si-CH_3), 1450 (rocking vibration -CH_2-), 1530, 1630, 1710, 2860, 2920, 3330 cm^{-1}.

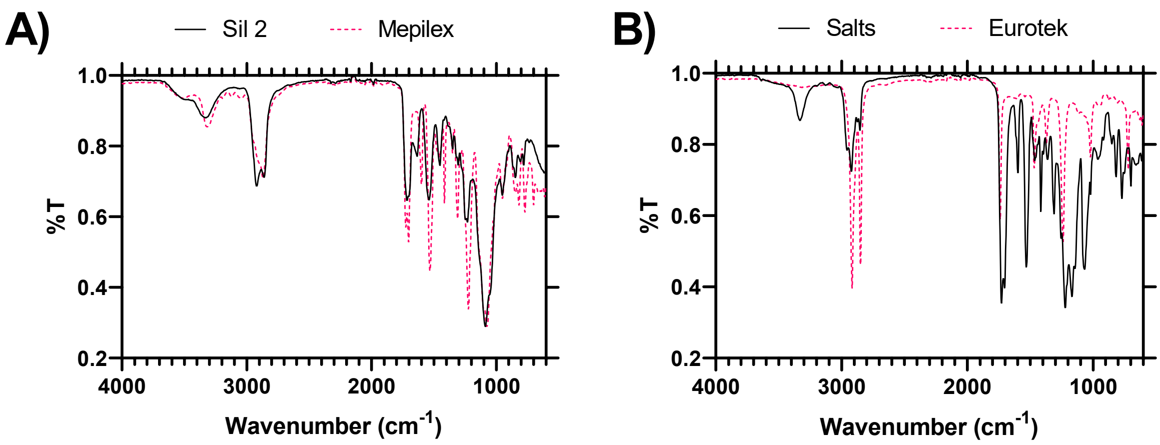

Figure S1 – FTIR Spectra of adhesive dressings of A) Silicone Adhesives and B) Hydrocolloid adhesives.

SEM

Of all the samples analysed the Sil2 was the most difficult to analyse via SEM as the material underwent a slow expansion when exposed to the vacuum required for imaging (**Figure S2**). For this reason sample analysis had to be carried out rapidly before the material expanded into an open foam structure (the surface visibly became rougher and stressed once removed from the chamber) and sections of the material appeared visibly damaged by the electron microscope at high levels of magnification (see **Figure S3**).

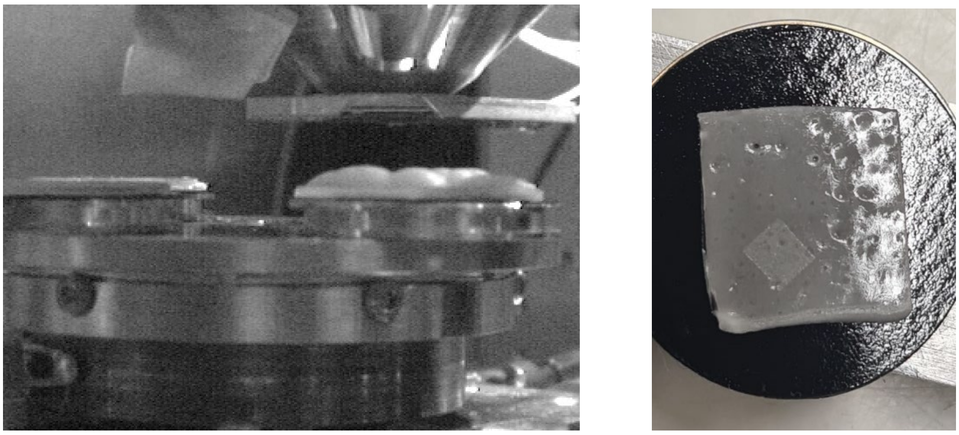

Figure S2 – Exemplar photographs of SEM. Left: novel silica expansion (bubbling) under vacuum. Right: damage to surface after exposure during electron microscopy analysis

As a result the SEM-EDX images presented in Figure 2 of the main manuscript are our best understanding of the natural material composition – however for clarity we have provided an EDX image of the expanded (swollen) foam which represents a much higher silicon content on the product surface as shown in **Figure S3**.

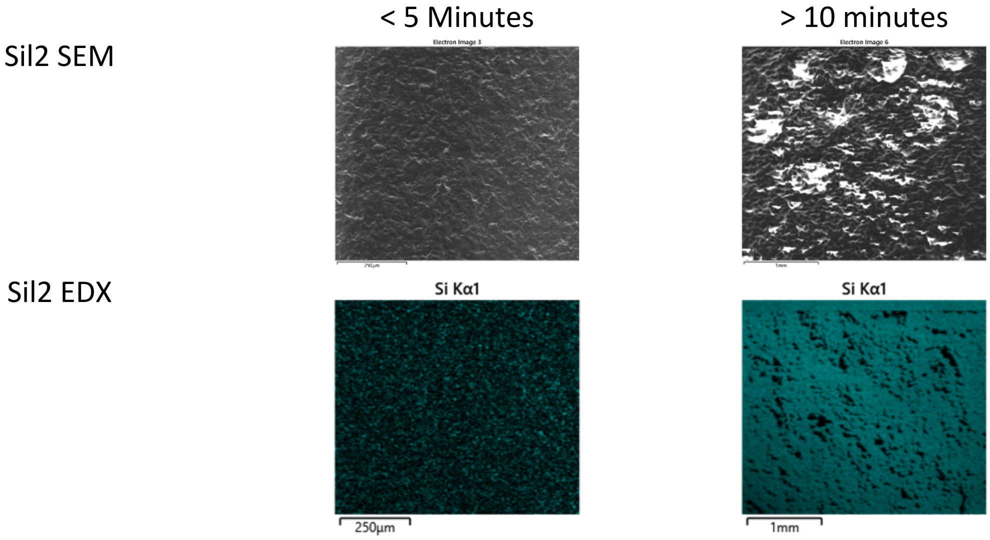

Figure S3 – Comparison of SEM and EDX (Sodium element mapping) profiles of novel silicone composite at initial, and after long exposure to chamber vacuum

Vapour Permeability

Vapour permeability measurements in the manuscript shows the vapour transmission through the medical dressings at ambient conditions, and at high humidity. Measurements were actually carried out after the samples were normalised for 1 hour in 5 different specified temperatures and humidities as shown below in **Figure S4**

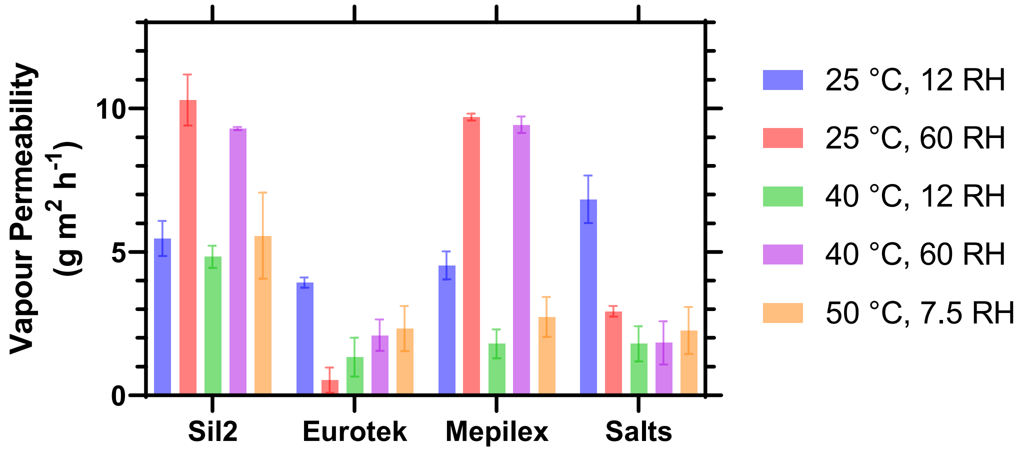

Figure S4 – Vapour permeability of 4 medical dressings after 1 hour normalisation in different environmental conditions (temperature in °C, % Relative Humidity)

Overall the humidity of the Sil2 and the Mepilex were higher than the hydrocolloid equivalents. Increasing the temperature had no significant impact on the vapour permeability measured, although it did increase as the sample ambient humidity was increased. This contradicts the silicone adhesives which showed decreasing vapour permeability with both temperature and relative humidity.

Swelling %

Swelling measurements were carried out on slices of each adhesive dressing by comparing specified temperatures and humidities as shown below in **Table S1** and **Figure S5**

Table S1 – Measurements of Swelling % measurement of dressing materials

		0 Hrs Bounding Box							24 Hrs Bounding Box							0 Hrs Outline					24 Hrs Outline					Bounding Box Difference			Outline Difference
**Cell**	**Material**	Label	Box Area	Mean	Min	Max	RRLength	RRWidth	Label	Box Area	Mean	Min	Max	RRLength	RRWidth	Label	Box Area	Mean	Min	Max	Label	Box Area	Mean	Min	Max	Area	Len	Wid	Pixel Area
A1	Sil2	0 A1.png	87149	166.961	96	255	374.425	232.75	24 A1.png	73465	176.343	96	254	353.604	226.449	0 A1.png	70730	183.433	98	255	24 A1.png	63546	188.884	113	254	84.30%	94.44%	97.29%	89.84%
A2	Sil2	0 A2.png	95533	178.107	96	255	408.251	234.196	24 A2.png	98952	173.555	96	255	399	248	0 A2.png	87029	186.13	99	255	24 A2.png	85541	185.706	107	255	103.58%	97.73%	105.89%	98.29%
A3	Sil2	0 A3.png	108749	172.403	96	255	387.312	284.242	24 A3.png	106199	163.568	96	255	371.15	287.873	0 A3.png	96270	182.307	103	255	24 A3.png	91662	174.284	113	255	97.66%	95.83%	101.28%	95.21%
A4	Sil2	0 A4.png	118789	161.779	96	255	363.697	326.617	24 A4.png	124796	162.257	96	255	377.808	332.043	0 A4.png	106237	169.55	103	255	24 A4.png	106461	173.666	97	255	105.06%	103.88%	101.66%	100.21%
A5	Sil2	0 A5.png	54680	176.71	96	255	301.33	181.46	24 A5.png	55678	162.856	96	255	302.152	184.277	0 A5.png	51070	182.415	99	255	24 A5.png	51117	168.821	110	255	101.83%	100.27%	101.55%	100.09%
A6	Sil2	0 A6.png	59204	172.19	96	255	317.932	186.216	24 A6.png	62157	172.154	96	255	321.316	193.446	0 A6.png	54689	178.48	98	255	24 A6.png	56175	180.262	117	255	104.99%	101.06%	103.88%	102.72%
B1	Eurotek	0 B1.png	83979	173.179	96	255	319.128	263.143	24 B1.png	92624	175.307	96	255	336.127	283.605	0 B1.png	76028	181.25	97	255	24 B1.png	82756	184.764	118	255	110.29%	105.33%	107.78%	108.85%
B2	Eurotek	0 B2.png	73049	159.675	95	255	276.788	263.931	24 B2.png	89753	170.676	96	255	305.489	297.718	0 B2.png	66330	166.125	95	255	24 B2.png	78007	181.92	123	255	122.87%	110.37%	112.80%	117.60%
B3	Eurotek	0 B3.png	74956	155.668	91	255	280.283	267.437	24 B3.png	84484	162.919	96	255	294.571	287.484	0 B3.png	65117	164.684	91	255	24 B3.png	72746	173.717	104	255	112.71%	105.10%	107.50%	111.72%
B4	Eurotek	0 B4.png	77990	171.004	96	255	281.322	277.229	24 B4.png	91473	172.076	96	255	308.351	296.66	0 B4.png	69582	180.066	104	255	24 B4.png	79741	183.269	96	255	117.29%	109.61%	107.01%	114.60%
B5	Eurotek	0 B5.png	95213	153.646	91	255	365.579	260.451	24 B5.png	110790	164.345	96	255	380.653	291.831	0 B5.png	88314	158.147	91	255	24 B5.png	99229	172.306	96	255	116.36%	104.12%	112.05%	112.36%
B6	Eurotek	0 B6.png	88289	152.281	86	255	334.531	264.933	24 B6.png	104854	155.562	96	255	350.79	299.902	0 B6.png	78358	159.414	86	255	24 B6.png	92548	163.478	96	255	118.76%	104.86%	113.20%	118.11%
C1	Mep	0 C1.png	114198	174.384	89	253	341.245	334.651	24 C1.png	121988	165.862	96	255	381.609	343.096	0 C1.png	84474	201.911	96	253	24 C1.png	94989	185.716	97	255	106.82%	111.83%	102.52%	112.45%
C2	Mep	0 C2.png	107205	176.283	96	255	373.789	293.438	24 C2.png	132872	166.443	96	255	405.242	330.68	0 C2.png	87970	193.826	96	255	24 C2.png	103835	186.143	96	255	123.94%	108.41%	112.69%	118.03%
C3	Mep	Lost Structural Integrity																											
C4	Mep	0 C4.png	122821	159.992	96	255	351.599	349.339	24 C4.png	122743	159.253	95	255	417.757	303.583	0 C4.png	85244	188.202	106	255	24 C4.png	95923	176.938	95	255	99.94%	118.82%	86.90%	112.53%
C5	Mep	0 C5.png	107703	165.769	96	255	360.83	303.991	24 C5.png	184530	143.492	96	255	502.194	367.447	0 C5.png	85492	183.895	105	255	24 C5.png	102688	181.339	96	255	171.33%	139.18%	120.87%	120.11%
C6	Mep	Lost Structural Integrity																											
D1	Salts	0 D1.png	76321	170.223	95	236	396.09	192.686	24 D1.png	72205	170.613	96	241	374.438	192.849	0 D1.png	70144	176.759	95	236	24 D1.png	65657	178.055	114	241	94.61%	94.53%	100.08%	93.60%
D2	Salts	0 D2.png	66636	170.404	96	255	321.283	207.406	24 D2.png	66765	170.754	96	255	323.209	206.571	0 D2.png	61413	176.731	120	255	24 D2.png	59399	180.025	121	255	100.19%	100.60%	99.60%	96.72%
D3	Salts	0 D3.png	82714	168.516	96	255	394.474	209.683	24 D3.png	78715	152.683	96	255	384.536	206.358	0 D3.png	72399	178.847	102	255	24 D3.png	66936	162.656	102	255	95.17%	97.48%	98.41%	92.45%
D4	Salts	0 D4.png	60263	176.915	96	255	281.295	214.117	24 D4.png	59669	172.382	96	255	279.276	213.656	0 D4.png	56871	181.732	123	255	24 D4.png	56649	176.454	113	255	99.01%	99.28%	99.78%	99.61%
D5	Salts	0 D5.png	82280	165.741	96	255	391.524	210.208	24 D5.png	66591	169.641	96	255	267.904	248.652	0 D5.png	71839	175.877	97	255	24 D5.png	61127	176.224	131	255	80.93%	68.43%	118.29%	85.09%
D6	Salts	0 D6.png	66329	166.958	96	255	324.89	204.158	24 D6.png	43664	179.672	96	255	264.337	165.172	0 D6.png	61248	172.845	119	255	24 D6.png	40325	186.6	125	255	65.83%	81.36%	80.90%	65.84%

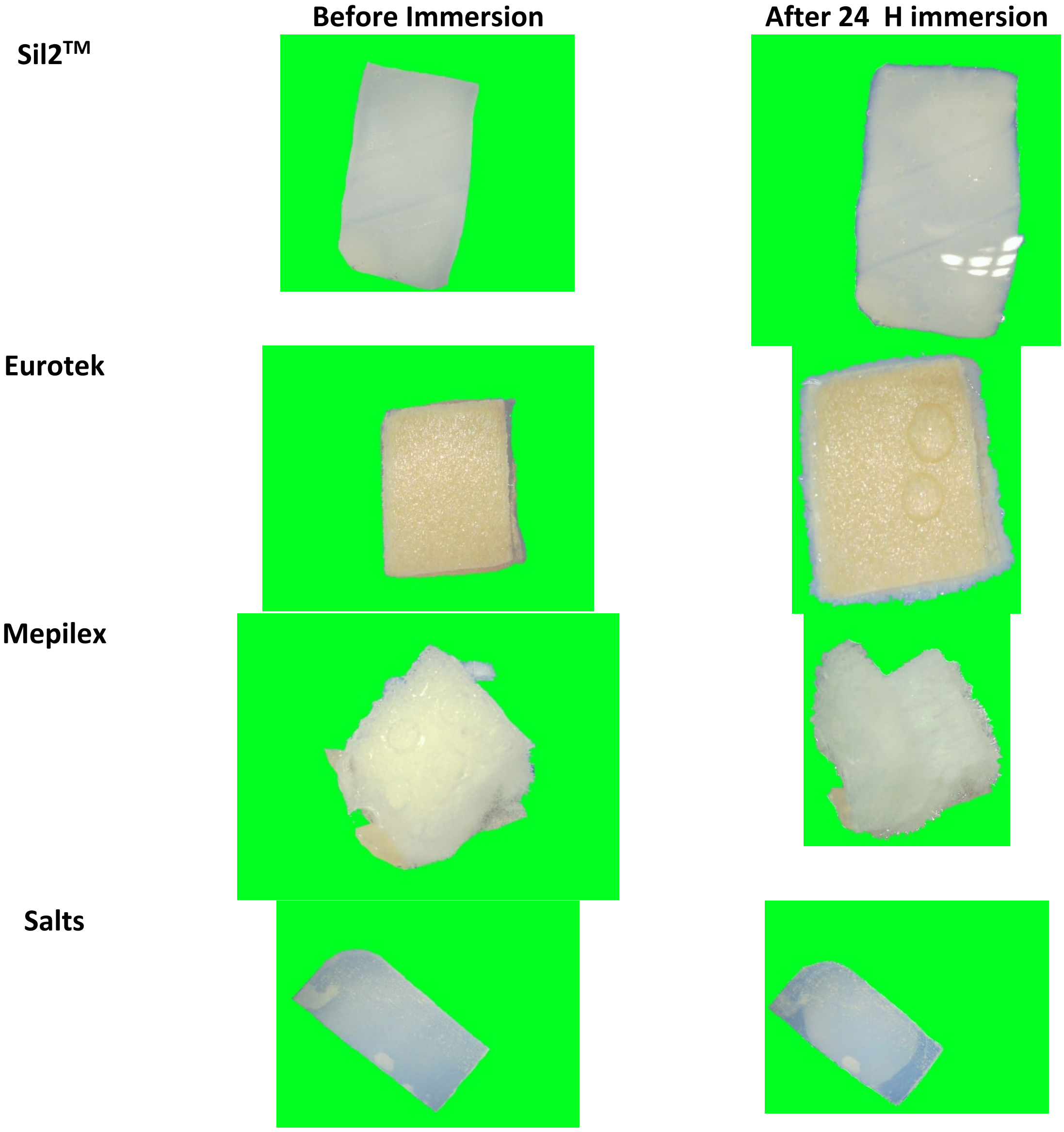

Figure S5 – Exemplar images of skin adhesive dressings swelling in aqueous suspension

2. Adhesive Dressing Moisture Management Comparisons

The relative hydrophobicity of the dressings were determined by comparing water contact angle measurements using a VCA Optima (AST Products, USA) camera. A liquid of water was deposited on the dressing and illuminated to determine the contact angle. This was repeated three times and an example image is shown in **Figure S6.**

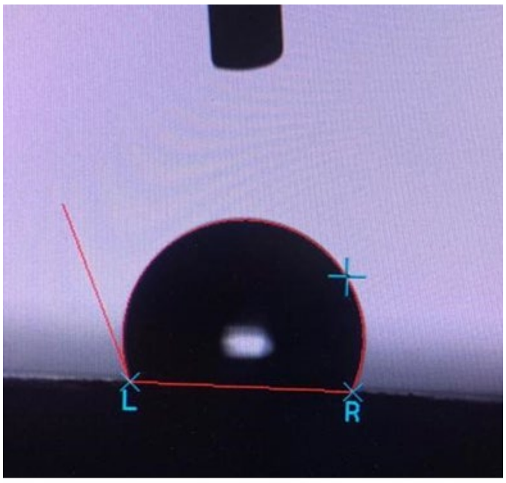

Figure S6 – Liquid droplet resting on hydrocolloid medical dressing.

The contact angle was determined to be 98 ± 5.0 for the hydrocolloid and 112 ± 1.3 for Sil2, showing a clear statistically significant difference between the two materials ($p < 0.0001$). However both dressing adhesive surfaces demonstrate overall hydrophobic properties – the silicone more than the hydrocolloid but it is not correct to claim one is hydrophilic and the other is hydrophobic. At best the hydrocolloid can be considered closer to neutral.

The relative vapour permeability of the dressings were measured after acclimatisation in a primitive skin model system – with the adhesive flange sandwiched between a PU sheet and Whatman filter paper as shown in **Scheme S1**. This was done to mimic the use of these adhesives on human skin where the upper PU film mimics the protective layer on top of a medical dressing, and the filter paper separates the adhesive from moisture sources. Below the lower barrier layer a standard piece of tissue paper was used, either wet or dry, in an experimental design created to mimic the skin surface where a high moisture content is separated from the adhesive by a thin dermal barrier. In this way it was intended we could observe changes in the material vapour permeability in the absence / presence of moisture vapour permeating through the system.

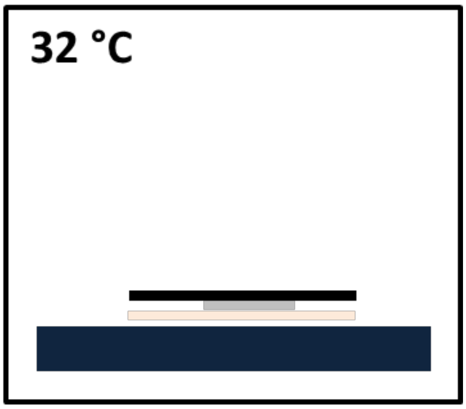

Scheme S1 – Adhesive sandwich test where the material is acclimatised at relative temperature and humidity

It is interesting to note that before we began this experiment we tested the relative occlusion of the PU Film used in the absence of an adhesive, interrogating how additional layers restricted the TEWL. Here we found that one layer of PU film is relatively permeable, further additions of the film, and that when the system was exposed to humidity a higher vapour loss was observed providing a statistically significant variation (P = 0.0001). Increasing layers of PU however reduced the effect as the upper layer (through which vapour measurements were carried out) were increasingly separated from the moisture source (average P = 0.0896). The data is shown in **Figure S7**.

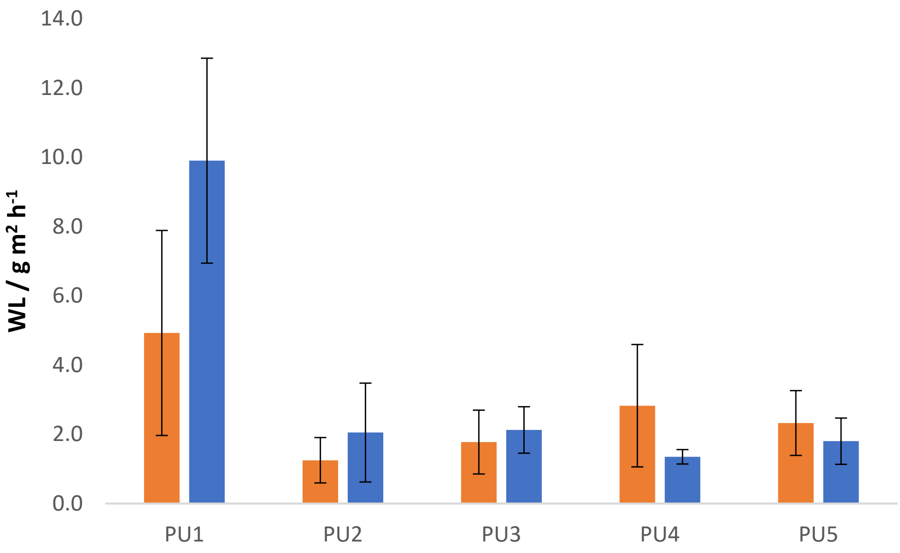

Figure S7 – Vapour Loss through 1 – 5 layers of PU when system was dry (orange) and in presence of moisture (blue).

Additionally there was no significant difference between the data measured after 1 or 4 hours acclimatisation (data not presented) and so future measurements were carried out after 1 hour acclimatisation.

The analysis of hydrocolloid and silicone materials was then carried out and produced the data shown in **Figure S8**.

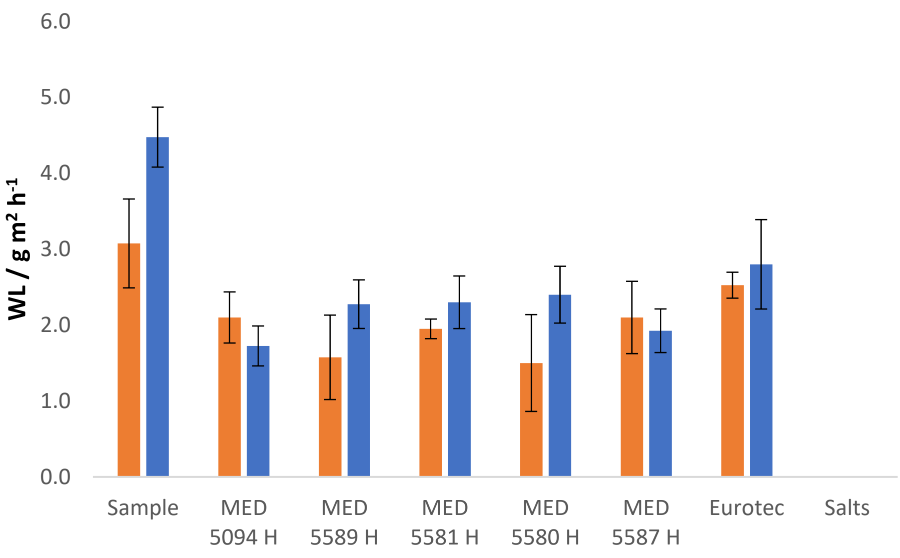

Figure S8- Analysis of Silicone and Hydrocolloid adhesive dressings vapour permeability when dry (orange) and in presence of moisture (blue).

3. Staining of Human Fibroblast Cells

Studies were undertaken of both the biocompatibility of the dressings and CC stain against human fibroblast cells. Initial compatibility measurements were carried out using human fibroblast cells donated by F45 (female aged 45 years old).

A visual cell count was performed both before, 1 hour following and 48 hours following 2 ml CC addition to an incubating cell suspension. The cells took up the majority of the dye within 1 hour of staining (**Figure S9 and Table S2**) in a uniform manner revealing on subcellular structures. Analysis of counted cells shows no statistical difference in cell numbers following staining ($p = 0.44$).

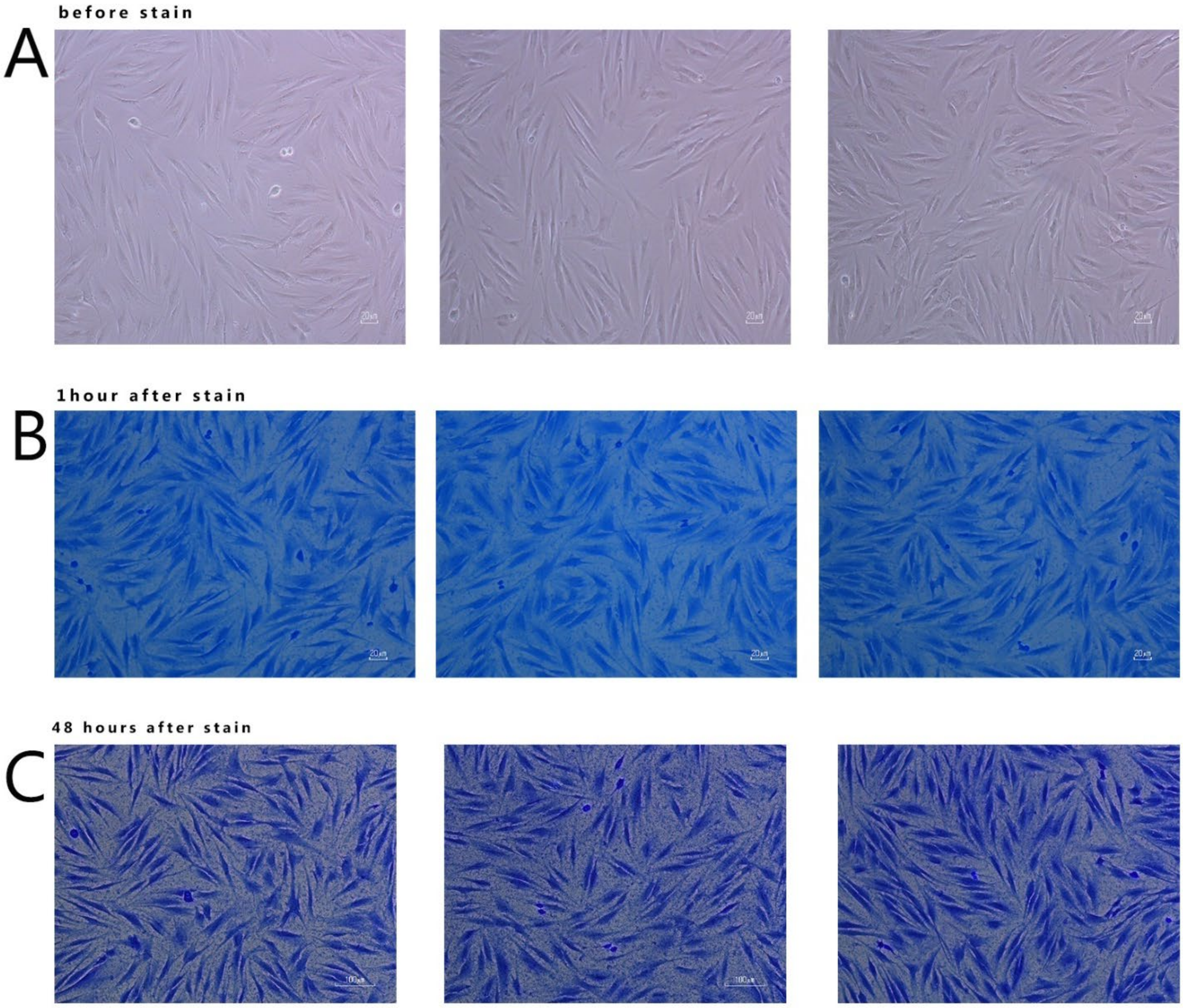

Figure S9 – images show fibroblast cells before and after being placed in a CC solution and left in an incubator. Incubator was set to 37 °C and 5% CO_2. (A) fibroblast cells adhered onto cell culture flask before staining with CC. (B) fibroblast cells left in 2ml of CC for an hour in the incubator. (C) Fibroblast cells left in 2ml of CC solution for 48 hours in the incubator.

Table S2 - Fibroblast cell counts from **Figure S10**

Colloidal Coomassie treatment	Cell count 1	Cell count 2	Cell count 3	Average cell count
No CC	139	150	161	150
1 Hour	144	131	140	138
48 Hours	151	152	163	155

Cells were also cultured on top of both hydrocolloid and silicone medical dressings.

Cell proliferation was measured using a WST-1 assay kit using a transwell plate.500 µl of WST 1 was added to each well, incubated at 37 °C with 5 % CO_2 for 4 hours. Absorbance was measured at 450 nm using a plate reader, determining the average absorbance from three repeats. A background reading at 690 nm was taken to subtract noise from the experiment. This data is shown in **Figure S10**.

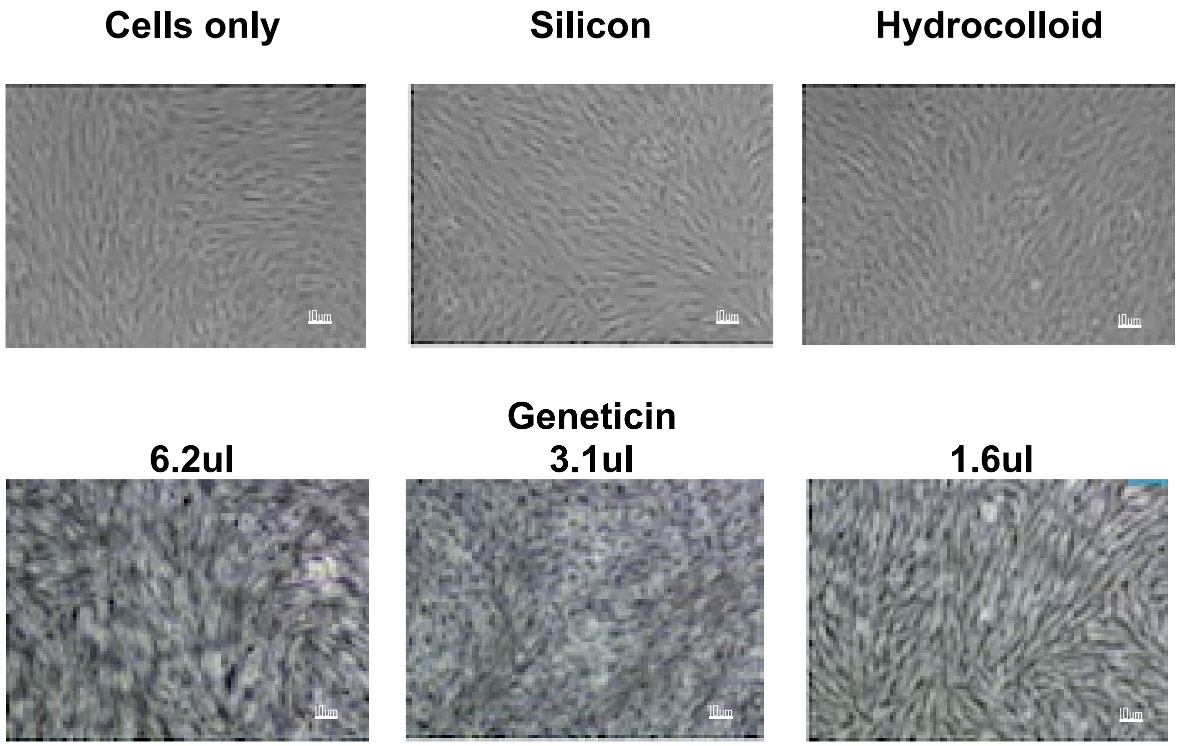

Figure S10 – Top: Images of human fibroblast cells under inverted light microscope (100 x) seeded onto culture plates, silicon medical dressings and hydrocolloid dressings. Bottom: Cells seeded with geneticin (6.2, 3.1 and 1.6 µl).

The final data, as shown in **Table S3**, indicates that cell proliferation was uninterrupted by the presence of either the silicone or hydrocolloid dressing, although a one-way ANOVA statistical test showed that the statistical difference between the cells alone, with silicone or

with hydrocolloid are not significantly different. This is as expected for a biocompatible product.

Table S3 – Absorption of WST-1 transwell plates following WST-1 Assay.

	Au	SD	N
Cells	0.1364	0.0136	3
Silicon	0.1667	0.0043	3
Hydrocolloid	0.1951	0.0209	3
Geneticin 6.3 ul	0.114	0.0005	3
Geneticin 3.1 ul	0.1006	0.0002	3
Geneticin 1.6 ul	0.0951	0.0007	3

Finally cells cultured on the hydrocolloid and silicone were stained using CC dye and the proliferation of protein on the dressing surface was compared after destaining, as shown in **Figure S11**. Unlike protein stains (where solutions of bovine serum albumin was added to the dressing) there was greater background adhesion of dye on the material surface, particularly for the hydrocolloid dressing.

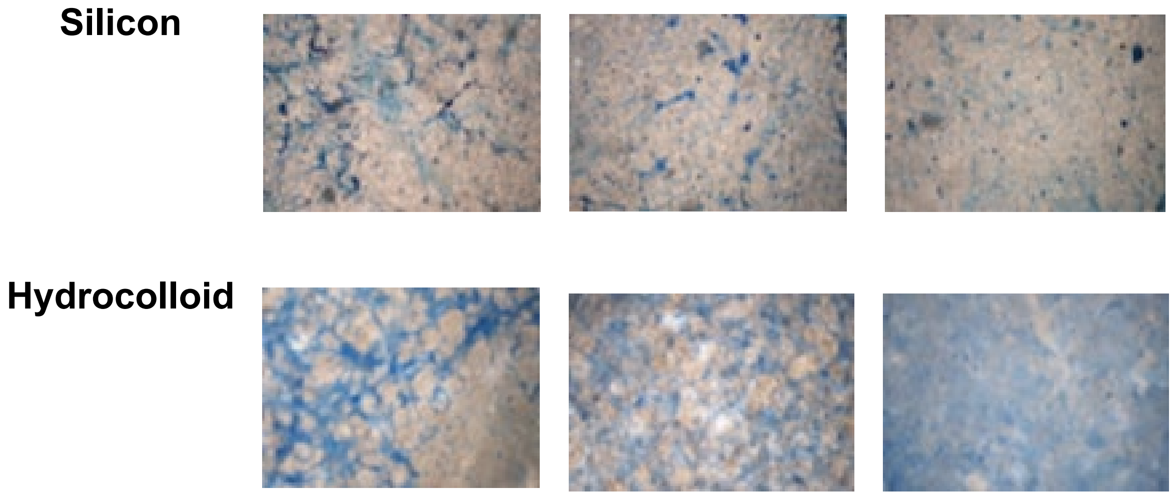

Figure S11 Images of human fibroblast cells on top of hydrocolloid and silicon medical dressings and stained with colloidal Coomassie under light microscope (x100).

4. TEWL ANOVA Data

The TEWL data from all participants was used in a 2-way ANOVA comparison between 6 and 24 hours are shown in **Table S4**.

Table S4. Summary of TEWL Data Across Entire Participant Study

		All			6 Hours			24 Hours		
		Mean	SD	N	Mean	SD	N	Mean	SD	N
Silicone 1	LA	4.25	2.33	31	3.82	1.52	18	4.84	3.11	13
	RA	5.07	3.76	31	4.79	2.27	18	5.45	5.28	13
	LT	6.07	3.00	31	6.46	3.26	18	5.52	2.61	13
	RT	5.44	2.74	31	6.18	2.74	18	4.42	2.49	13
Hydrocolloid 1	LA	2.06	1.27	31	1.68	1.00	18	2.58	1.45	13
	RA	2.35	1.58	31	1.98	0.95	18	2.86	2.12	13
	LT	3.01	1.39	31	3.00	1.53	18	3.03	1.24	13
	RT	2.67	1.29	31	2.61	1.49	18	2.75	0.99	13
Skin 1	LA	5.84	2.38	31	6.02	2.17	18	5.58	2.71	13
	RA	5.52	2.43	31	6.00	1.54	18	4.85	3.26	13
	LT	7.31	4.55	31	8.18	5.22	18	6.11	3.24	13
	RT	7.33	4.71	31	8.12	5.41	18	6.23	3.43	13
Silicone 2	LA	2.63	1.78	30	2.42	1.80	17	2.90	1.79	13
	RA	3.02	1.99	25	3.28	2.28	15	2.63	1.46	10
	LT	4.54	3.24	24	5.26	3.65	16	3.10	1.54	8
	RT	4.87	3.03	22	5.12	3.27	15	4.31	2.58	7
Hydrocolloid 2	LA	2.33	1.63	30	1.97	1.31	18	2.88	1.96	12
	RA	2.47	1.23	31	2.32	1.27	18	2.68	1.20	13
	LT	2.93	1.24	30	2.83	1.18	18	3.07	1.38	12
	RT	3.01	1.29	31	2.77	1.26	18	3.35	1.30	13
Skin 2	LA	5.89	3.69	23	6.08	4.07	17	5.35	2.49	6
	RA	6.74	2.74	24	7.99	2.14	17	3.71	1.21	7
	LT	8.19	4.25	24	8.90	4.26	17	6.46	3.97	7
	RT	8.01	5.22	24	8.73	5.49	17	6.24	4.34	7
Under Silicone	LA	8.60	3.82	18	7.30	2.58	8	9.64	4.44	10
	RA	9.58	3.96	16	10.55	4.32	6	9.00	3.84	10
	LT	13.35	4.08	17	12.45	2.42	8	14.14	5.17	9
	RT	13.98	5.59	16	16.40	5.68	8	11.55	4.62	8
Under Hydrocolloid	LA	19.27	6.10	20	16.26	3.86	8	21.28	6.63	12
	RA	20.54	7.45	21	20.78	7.77	8	20.40	7.56	13
	LT	22.25	9.37	20	20.75	11.81	8	23.25	7.76	12
	RT	24.33	8.41	21	22.56	6.87	8	25.42	9.32	13

5. Patch Loss Rate

The study was designed so that across 30 participants there would be 124 patches of Sil2 and hydrocolloid applied respectively, with 62 on forearms and 62 on torso. After the study was complete 101 of 124 silicone patches had remained in place whilst 122 of the hydrocolloids had remained. The loss of Sil2 was found to be 89% retention on the forearms and 75% retention on the torso.

In preparation of this study Sil2 was cut into smaller strips (2 – 3 cm square) – and it was observed by participants who lost patches that the slow peeling from the edge led to it detaching entirely with just a few knocks. This implies a correlation between patch size (surface area of adhesion) and sample stability. Unfortunately this was noticed too late for the patch sizes to be increased for the full participant trial to be altered.

Only patches that retained in place to the end of the participant trial are counted in the data presented in the manuscripts, and samples that detached early were noted and then discarded from future analysis.

6. Peel Adhesion Tests

Peel test measurements were recorded via multiple measurements where the adhesive was pulled at a constant rate vertically upwards away from participant skin as shown in **Figure S12**

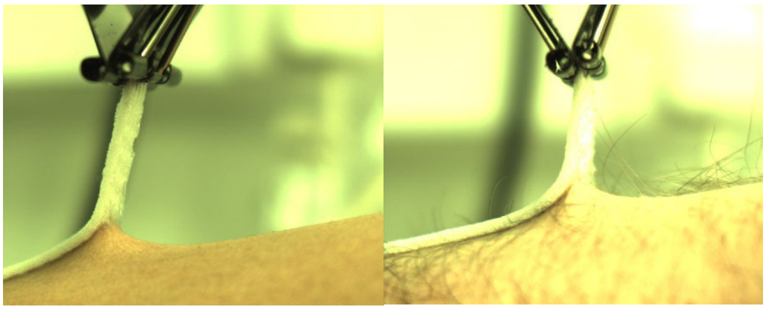

Figure S12 – Exemplar images of adhesive peeling from participant skin

Raw data for peel strength measurements are shown in **Figure S13** – each line represents a different participant tested. Participants wore strips for 3 - 6 hours prior to removal.

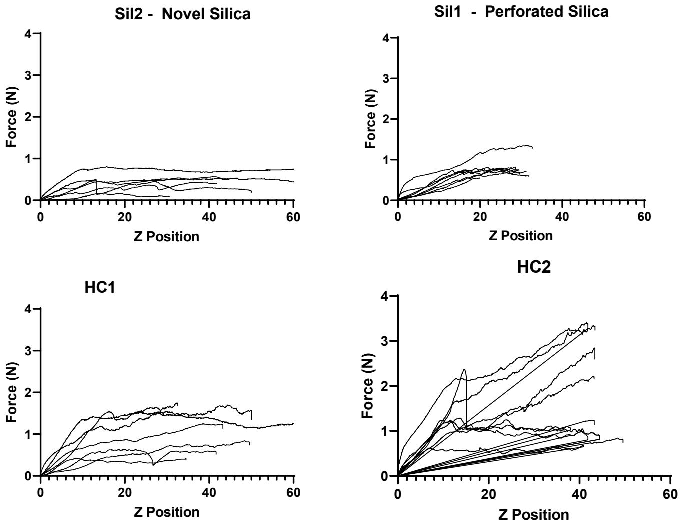

Figure S13 – Raw Force (resistance) of removal (Z position of clamp) curves of hydrocolloid and silicon dressings (each line represents a distinct individual).

Table S5 – Novel Silicone Dressings

	1	2	3	4	5	6
Peak load	0.44	0.456536	0.56954	0.501738	0.526044	0.807523
Peak position	38.86	27.1998	41.2789	12.978	42.2991	15.7577
Peak Peel Adhesion	0.034	0.045654	0.071193	0.045613	0.026302	0.040376
SS start pos	35	32	35	14	11	11
SS stop pos	39	49	45	30	79	79
SS average Peel Force	0.43	0.273959	0.534409	0.121609	0.485373	0.727514
SS Average Peel Adhesion	0.033	0.027396	0.066801	0.011055	0.024269	0.036376

Table S6 – Mepilex Perforated Silica Adhesive

	1	2	3	4	5	6	7
Peak load	0.70	0.81	0.72	0.78	0.56	0.82	0.77
Peak position	27.00	21.76	24.78	27.50	19.86	28.52	24.90
Peak Peel Strength	0.074	0.081	0.068	0.078	0.059	0.087	0.081
SS start pos	20	15	13	23	18	19	17
SS stop pos	31.5	29	30	29	19.9	28	29
SS average Peel Force	0.63	0.74	0.66	0.76	0.54	0.77	0.72
SS Average Peel Strength	0.066	0.074	0.063	0.076	0.057	0.081	0.076

Table S7 – Salts Hydrocolloid Adhesive

	1	2	3	4	5	6	7
Peak load	0.4158	0.64186	0.84075	1.24756	1.75382	1.70272	1.53198
Peak position	8.1598	18.518	48.335	38.3779	32.3177	44.398	22.6387
Peak Peel Strength	0.04620	0.04937	0.0420	0.05940	0.08769	0.08513	0.07659
SS start pos	21	17	31	38	26	11	11
SS stop pos	45	24	49	43	32	79	79
SS average Peel Force	0.34800	0.61682	0.7365	1.23259	1.63183	1.40157	1.39827
SS Average Peel Strength	0.03866	0.04744	0.0368	0.05869	0.08159	0.07007	0.0699

Table S8 – Eurotek Hydrocolloid Adhesive

	1	2	3	4	5	6	7	8
Peak load	3.34	2.22	1.21	1.24	2.85	3.41	2.85	1.26
Peak position	43.01	43.03	11.32	42.36	43.33	44.40	0.00	45.48
Peak Peel Strength	0.334	0.222	0.121	0.124	0.285	0.170	0.285	0.126
SS start pos	37		14	8			16	11
SS stop pos	43		40	40			40	79
SS average Peel Force	3.24		1.05	0.57			0.94	0.95
SS Average Peel Strength	0.324		0.105	0.057			0.094	0.095

7. TEWL Variability

In order to understand the variability of TEWL measurements across an individual sample two volunteers (1M, 1F) recorded their TEWL regularly over a three / two week period, noting the time and their activity levels before each measurement. The data from this was then used to estimate the variability in TEWL depending on environmental conditions. The volunteers both had a clear distinction in the TEWL of their upper arms (M average 6.75 ± 1.33, F average 5.33 ± 1.79) compared to their torso (M: 3.75 ± 0.98, F: 4.73 ± 1.20). This is not a trend unique to this individual and is repeated commonly across participants. Of particular interest to us was the variability / change in TEWL measurements over time.

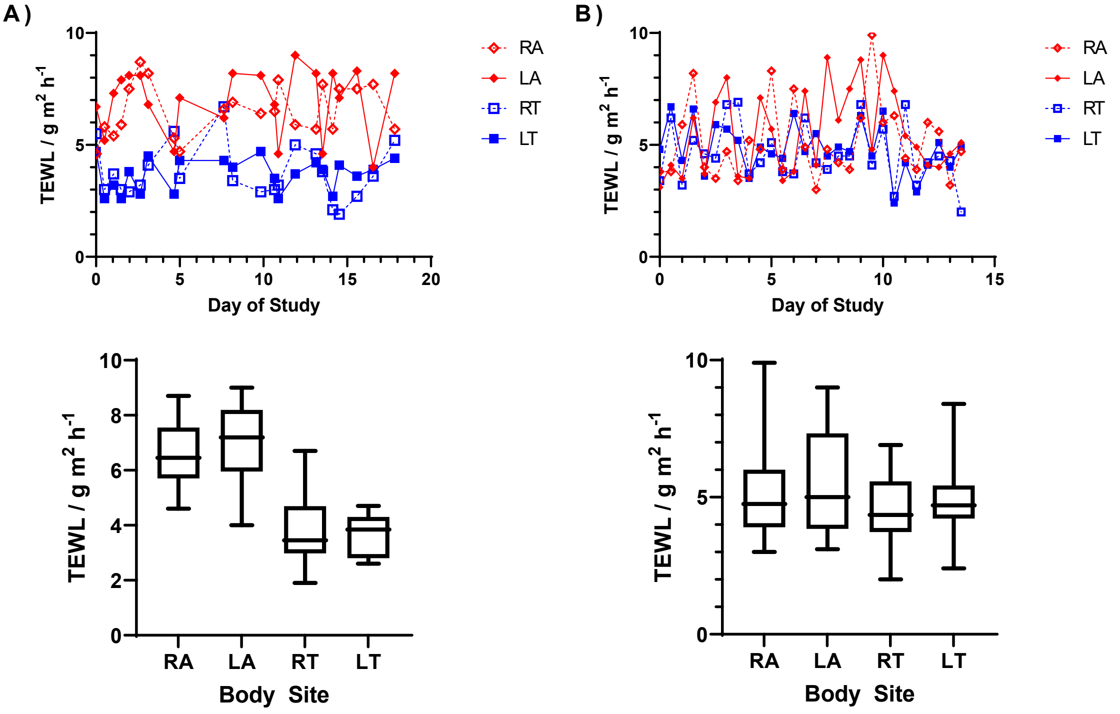

Figure S14 – TEWL variability data for A) 1M and B) 1F volunteers across 3 weeks. Top: TEWL measurements across 3 week period, Bottom: Average and std. deviation of TEWL in each body part.

A 2-way ANOVA was carried out on the TEWL data which showed that there was no significant to the date/time that the measurements were taken, or the activity level of the participant beforehand, (P = 0.76) but there was a significant difference between the four body sites (P < 0.0001). T test comparisons of the arms (LA and RA) showed no significant in the datasets (P = 0.3) whilst comparing arms to torso showed significant differences in the male but not the female (M: P<0.001, F: P ≈ 0.04). This indicates that whilst forearm and torso skin can be significantly different both are far more variable within the individual depending on factors unrecorded in the study.

8. Stain Analysis

Colloidal Coomassie staining imparted a clear blue stain on the protein adhered to the adhesive surface as shown in **Figure S15.**

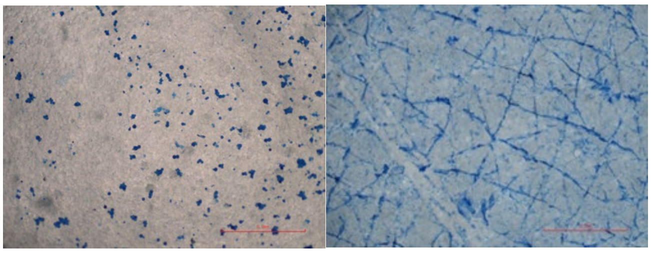

Figure S15 – Optical microscope image of colloidal Coomassie protein stained silicone (left) and hydrogel (right) recovered from participant left forearm.

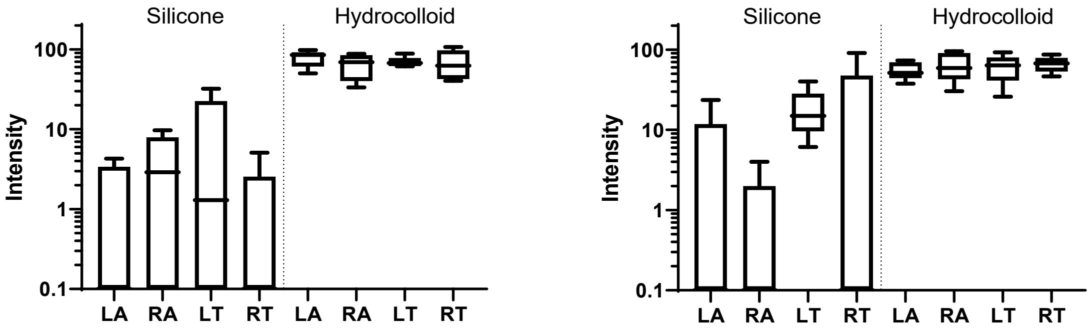

Figure S16 – Protein stain intensity on silicone and hydrocolloid dressings from male (left) and female (right) participants

9. Uniaxial Mechanical Testing

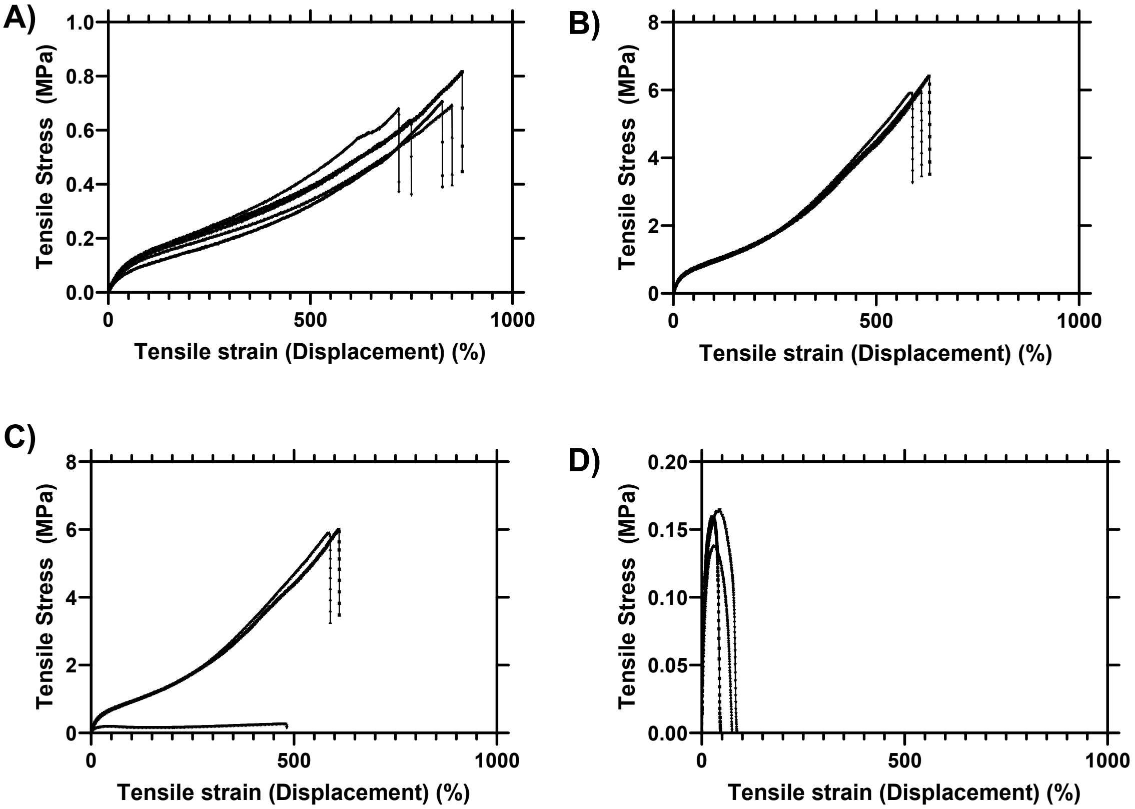

Figure S17 – Stress / Strain curves for mechanical measurements depicted in Table 4 for Sil2 (A), Salts (B), Eurotek (C) and Eurotek Hydrocolloid alone (D)

ESI References

1. Salih, S. I., Oleiwi, J. K. & Ali, H. M. Study the Mechanical Properties of Polymeric Blends (SR/PMMA) Using for Maxillofacial Prosthesis Application. *Int. Conf. Mater. Eng. Sci.* **454**, (2018).
